# An orally available P1′-5-fluorinated M^pro^ inhibitor blocks SARS-CoV-2 replication without booster and exhibits high genetic barrier

**DOI:** 10.1093/pnasnexus/pgae578

**Published:** 2025-01-07

**Authors:** Nobuyo Higashi-Kuwata, Haydar Bulut, Hironori Hayashi, Kohei Tsuji, Hiromi Ogata-Aoki, Maki Kiso, Nobutoki Takamune, Naoki Kishimoto, Shin-ichiro Hattori, Takahiro Ishii, Takuya Kobayakawa, Kenta Nakano, Yukiko Shimizu, Debananda Das, Junji Saruwatari, Kazuya Hasegawa, Kazutaka Murayama, Yoshikazu Sukenaga, Yuki Takamatsu, Kazuhisa Yoshimura, Manabu Aoki, Yuri Furusawa, Tadashi Okamura, Seiya Yamayoshi, Yoshihiro Kawaoka, Shogo Misumi, Hirokazu Tamamura, Hiroaki Mitsuya

**Affiliations:** Department of Refractory Viral Diseases, National Center for Global Health and Medicine Research Institute, 1-21-1 Toyama, Shinjuku-ku, Tokyo 162-8655, Japan; Experimental Retrovirology Section, HIV and AIDS Malignancy Branch, National Cancer Institute, NIH, Bethesda, MD 20892, USA; Department of Infectious Diseases, International Research Institute of Disaster Science, Tohoku University, Aoba-ku, Sendai 980-8575, Japan; Department of Medicinal Chemistry, Institute of Biomaterials and Bioengineering, Tokyo Medical and Dental University, Chiyoda-ku, Tokyo 101-0062, Japan; Department of Refractory Viral Diseases, National Center for Global Health and Medicine Research Institute, 1-21-1 Toyama, Shinjuku-ku, Tokyo 162-8655, Japan; Department of Clinical Sciences, Kumamoto University Hospital, Chuo-ku, Kumamoto 860-8556, Japan; Division of Hematopoiesis, Joint Research Center for Human Retrovirus Infection & Graduate School of Medical Sciences, Kumamoto University, Chuo-ku, Kumamoto 860-0811, Japan; Division of Virology, Institute of Medical Science, University of Tokyo, Minato-ku, Tokyo 108-8639, Japan; Department of Environmental and Molecular Health Sciences, Faculty of Life Sciences, Kumamoto University, 5-1 Oe-honmachi, Chuo-ku, Kumamoto 862-0973, Japan; Department of Environmental and Molecular Health Sciences, Faculty of Life Sciences, Kumamoto University, 5-1 Oe-honmachi, Chuo-ku, Kumamoto 862-0973, Japan; Department of Refractory Viral Diseases, National Center for Global Health and Medicine Research Institute, 1-21-1 Toyama, Shinjuku-ku, Tokyo 162-8655, Japan; Department of Medicinal Chemistry, Institute of Biomaterials and Bioengineering, Tokyo Medical and Dental University, Chiyoda-ku, Tokyo 101-0062, Japan; Department of Medicinal Chemistry, Institute of Biomaterials and Bioengineering, Tokyo Medical and Dental University, Chiyoda-ku, Tokyo 101-0062, Japan; Department of Laboratory Animal Medicine, National Center for Global Health and Medicine Research Institute, 1-21-1 Toyama, Shinjuku-ku, Tokyo 162-8655, Japan; Department of Laboratory Animal Medicine, National Center for Global Health and Medicine Research Institute, 1-21-1 Toyama, Shinjuku-ku, Tokyo 162-8655, Japan; Experimental Retrovirology Section, HIV and AIDS Malignancy Branch, National Cancer Institute, NIH, Bethesda, MD 20892, USA; Division of Pharmacology and Therapeutics, Graduate School of Pharmaceutical Sciences, Kumamoto University, Chuo-ku, Kumamoto 862-0973, Japan; Structural Biology Division, Japan Synchrotron Radiation Research Institute, 1-1-1 Kouto, Sayo, Hyogo 679-5198, Japan; Graduate School of Biomedical Engineering, Tohoku University, Miyagi 980-8579, Japan; Department of Refractory Viral Diseases, National Center for Global Health and Medicine Research Institute, 1-21-1 Toyama, Shinjuku-ku, Tokyo 162-8655, Japan; Department of Refractory Viral Diseases, National Center for Global Health and Medicine Research Institute, 1-21-1 Toyama, Shinjuku-ku, Tokyo 162-8655, Japan; Tokyo Metropolitan Institute of Public Health, Shinjuku-ku, Tokyo 169-0073, Japan; Department of Refractory Viral Diseases, National Center for Global Health and Medicine Research Institute, 1-21-1 Toyama, Shinjuku-ku, Tokyo 162-8655, Japan; Department of Medical Technology, Kumamoto Health Science University, 325 Izumimachi, Kita-ku, Kumamoto 861-5598, Japan; Division of Virology, Institute of Medical Science, University of Tokyo, Minato-ku, Tokyo 108-8639, Japan; The Research Center for Global Viral Diseases, National Center for Global Health and Medicine Research Institute, 1-21-1 Toyama, Shinjuku-ku, Tokyo 162-8655, Japan; Department of Laboratory Animal Medicine, National Center for Global Health and Medicine Research Institute, 1-21-1 Toyama, Shinjuku-ku, Tokyo 162-8655, Japan; Division of Virology, Institute of Medical Science, University of Tokyo, Minato-ku, Tokyo 108-8639, Japan; The Research Center for Global Viral Diseases, National Center for Global Health and Medicine Research Institute, 1-21-1 Toyama, Shinjuku-ku, Tokyo 162-8655, Japan; Division of Virology, Institute of Medical Science, University of Tokyo, Minato-ku, Tokyo 108-8639, Japan; The Research Center for Global Viral Diseases, National Center for Global Health and Medicine Research Institute, 1-21-1 Toyama, Shinjuku-ku, Tokyo 162-8655, Japan; Department of Pathobiological Sciences, School of Veterinary Medicine, Influenza Research Institute, University of Wisconsin-Madison, Madison, WI 53711, USA; Department of Environmental and Molecular Health Sciences, Faculty of Life Sciences, Kumamoto University, 5-1 Oe-honmachi, Chuo-ku, Kumamoto 862-0973, Japan; Department of Medicinal Chemistry, Institute of Biomaterials and Bioengineering, Tokyo Medical and Dental University, Chiyoda-ku, Tokyo 101-0062, Japan; Department of Refractory Viral Diseases, National Center for Global Health and Medicine Research Institute, 1-21-1 Toyama, Shinjuku-ku, Tokyo 162-8655, Japan; Experimental Retrovirology Section, HIV and AIDS Malignancy Branch, National Cancer Institute, NIH, Bethesda, MD 20892, USA; Department of Clinical Sciences, Kumamoto University Hospital, Chuo-ku, Kumamoto 860-8556, Japan

**Keywords:** COVID-19, SARS-CoV-2, M^pro^ inhibitor, fluorine-scanning, genetic barrier

## Abstract

We identified a 5-fluoro-benzothiazole-containing small molecule, TKB272, through fluorine-scanning of the benzothiazole moiety, which more potently inhibits the enzymatic activity of SARS-CoV-2's main protease (M^pro^) and more effectively blocks the infectivity and replication of all SARS-CoV-2 strains examined including Omicron variants such as SARS-CoV-2^XBB1.5^ and SARS-CoV-2^EG.5.1^ than two M^pro^ inhibitors: nirmatrelvir and ensitrelvir. Notably, the administration of ritonavir-boosted nirmatrelvir and ensitrelvir causes drug–drug interactions warranting cautions due to their CYP3A4 inhibition, thereby limiting their clinical utility. When orally administered, TKB272 blocked SARS-CoV-2^XBB1.5^ replication without ritonavir in B6.Cg-Tg(K18-hACE2)2-Prlmn/J-transgenic mice, comparably as did ritonavir-boosted nirmatrelvir. When the ancestral SARS-CoV-2 was propagated with nirmatrelvir in vitro, a highly nirmatrelvir-resistant E166V-carrying variant (SARS-CoV-2^E166V−P14^) readily emerged by passage 14; however, when propagated with TKB272, no variants emerged by passage 25. SARS-CoV-2_E166V_ showed some cross-resistance to TKB272 but was substantially sensitive to the compound. X-ray structural analyses and mass-spectrometric data showed that the E166V substitution disrupts the critical dimerization-initiating Ser1′-E166 interactions, thereby limiting nirmatrelvir's M^pro^ inhibition but that TKB272 nevertheless forms a tight binding with M^pro^'s catalytic active sight even in the presence of the E166V substitution. TKB272 shows no apparent genotoxicity as tested in the micro-Ames test. Highly potent TKB272 may serve as a COVID-19 therapeutic, overcome resistance to existing M^pro^ inhibitors.

**Significance Statement**

Ritonavir-boosted nirmatrelvir targeting SARS-CoV-2's main protease (M^pro^) shows efficacy in patients with mild-to-moderate COVID-19. However, ritonavir causes drug–drug interactions that warrant cautions due to CYP3A4 inhibition, thus limiting nirmatrelvir's clinical utility. We newly identified a novel M^pro^ inhibitor, TKB272, which achieves higher intracellular concentrations, more potently inhibits M^pro^, and more effectively blocks the infectivity and replication of SARS-CoV-2 strains than nirmatrelvir. When orally administered in mice, TKB272 potently blocked SARS-CoV-2^XBB1.5^ without ritonavir. When propagated with nirmatrelvir in vitro, highly nirmatrelvir-resistant E166V-carrying SARS-CoV-2_E166V_ readily emerged; however, when propagated with TKB272, no variants emerged. TKB272 showed substantial activity against SARS-CoV-2_E166V_. Highly potent TKB272 may serve as a COVID-19 therapeutic and overcome resistance to existing M^pro^ inhibitors.

## Introduction

Although the waning acute phase of the COVID-19 pandemic has arrived and the World Health Organization ended its public health emergence of international concern (PHEIC) declaration on May 2023, concerns are present on the emergence of new variants (https://covid.cdc.gov/covid-data-tracker/#variant-proportions). It is also noteworthy that residual SARS-CoV-2 can persist in patients who have recovered from mild COVID-19, and that there is a significant association between viral persistence and long COVID symptoms (1). Currently, only three anti-SARS-CoV-2 therapeutics: remdesivir (2), molnupiravir (3), and nirmatrelvir (4), have been clinically available in the United States and other countries, and these three anti-SARS-CoV-2 therapeutics have been shown to provide clinical benefits (5–7). While there have been SARS-CoV-2 rebounds following the 5-day regimen treatment with nirmatrelvir/ritonavir, a most recent literature shows that the SARS-CoV-2 rebound has been observed in both nirmatrelvir-treated and -untreated individuals in randomized, double-blind, placebo-controlled trials and that the virologic rebound was not associated with COVID-19-related hospitalization or death (8). Other groups have also shown similar results (9, 10). It is unclear if the viral rebound represents the resumption of viral replication due to incompletely controlled infection due to inadequate length of therapy (5 days) or a natural biphasic pattern of viral replication, or else, more studies need to be done to figure out the mechanistic of the rebound. However, there are a few recent reports that nirmatrelvir-resistant variants carrying E166V/L50V substitutions, etc. emerge in immunocompromised individuals treated with nirmatrelvir/ritonavir therapy at low frequencies and transiently, presently posing only a low risk in the communities (11, 12). These current situations strongly suggest that the development of more potent, safe, and more tolerable therapeutics for COVID-19 is desperately required.

We have designed and synthesized approximately 700 different M^pro^ inhibitors, and have determined potent ones, including 5 h, TKB245, and TKB248, which have proved to be reversible-covalent M^pro^ inhibitors (13, 14). Here, we identified an orally available 5-fluoro-benzothiazole-containing small molecule, TKB272, through fluorine-scanning of the benzothiazole moiety which more potently inhibits the enzymatic activity of SARS-CoV-2's main protease (M^pro^) and more effectively block the infectivity and replication of SARS-CoV-2 variants than nirmatrelvir (4), molnupiravir (2), and ensitrelvir (15). When TKB272 was orally administered in SARS-CoV-2-susceptible mice, it blocked the replication of the Omicron variant SARS-CoV-2^XBB1.5^ without requiring ritonavir, more effectively than the extent nirmatrelvir did in combination with ritonavir.

## Results

### Identification of a 5-fluoro-benzothiazole-containing small molecule, TKB272, through fluorine-scanning

Fluorination of certain compounds often enhances their lipophilicity, cell membrane penetration, and oral bioavailability, since the carbon-fluorine bond is chemically more stable and more hydrophobic than the carbon–hydrogen bond (16–20). In the present study, based on the antiviral and structural features of a benzothiazole-containing M^pro^ inhibitor (13, 14, 21, 22), we attempted to optimize the antiviral activity of various TKB245-related congeners through fluorine-scanning and determined their virological, enzymological, and pharmacological features. When we determined the inhibitory activity of a previously reported 4-fluoro-benzothiazole-containing TKB245 (14) against M^pro^ of the ancestral SARS-CoV-2^WK-521^, its inhibition proved to be potent with its IC_50_ value of 0.0012 µM. However, a newly generated TKB272, which contains a fluorine atom at 5-position in the benzothiazole moiety, exhibited furthermore potent inhibition of the enzymatic activity of M^pro^s from SARS-CoV-2^WK-521^ with its IC_50_ value of 0.0007 µM (Table 1). Furthermore, TKB272's target specificity was confirmed when we found that none of the human cysteine enzymes, cathepsin L or calpain, were appreciably inhibited by the compound (Fig. S1). When a fluorine atom was annexed to 6- and 7-positions of benzothiazole, generating TKB273 and TKB252, their inhibition of M^pro^s from SARS-CoV-2^WK-521^ was limited (IC_50_ values = 0.0089 and 0.20 µM, respectively). When anti-SARS-CoV-2 activity of TKB272 was examined in cell-based assays using two different target cells (VeroE6 and HeLa^hACE2-TMPRSS2^ cells), its antiviral activity was significantly more potent than TKB245 (EC_50_ values of TKB272 against SARS-CoV-2^WK-521^ in VeroE6 and SARS-CoV-2^BQ.1.1^ in HeLa^hACE2-TMPRSS2^ = 0.007 and 0.0026 µM; those of TKB245 = 0.02 and 0.038 µM, respectively). Antiviral activity of TKB273 and TKB252 was much less potent with EC_50_ values of TKB273 against SARS-CoV-2^WK-521^ in VeroE6 and SARS-CoV-2^BQ.1.1^ in HeLa^hACE2-TMPRSS2^ = 0.043 and 0.114 µM; those of TKB252 = 0.062 and 0.057 µM, respectively. As compared with two clinically available M^pro^ inhibitors, nirmatrelvir and ensitrelvir, TKB272 was significantly more potent (EC_50_ values of nirmatrelvir against SARS-CoV-2^WK-521^ in VeroE6 and SARS-CoV-2^BQ.1.1^ in HeLa^hACE2-TMPRSS2^ = 0.77 and 0.051 µM. All compounds evaluated in this study showed no significant cytotoxicity (Table 1). In addition, in primary human nasal epithelial (HNEp) cells, TKB272 exerted more potent anti-SARS-CoV-2^Omicron EG.5.1^ activity (EC_50_: 0.031 µM) than that of nirmatrelvir (EC_50_: 11.1 µM) (Fig. S2).

**Table 1.** Fluorine scanning of TKB compounds.

Compound	Structure	SARS-CoV-2 M^pro^ enzyme assay IC_50_ (µM)	SARS- CoV-2^WK-521^ in VeroE6 cells EC_50_ (µM)	SARS-CoV-2^BQ.1.1^ in HeLa^hACE2-TMPRSS2^ cells EC_50_ (µM)	Cytotoxicity of compounds CC_50_ (µM)
TKB245 M.W. 653.6936	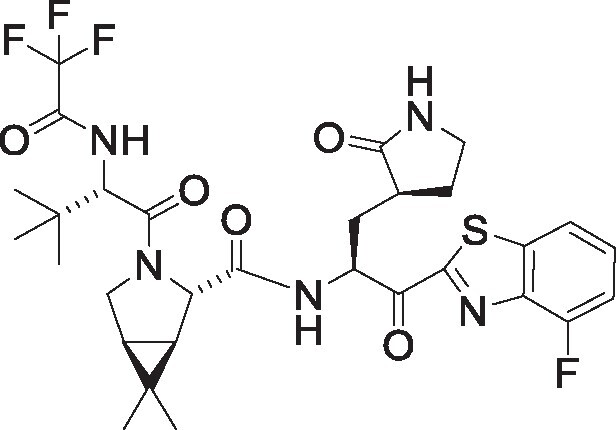	0.0012 ± 0.0004	0.020 ± 0.007	0.038 ± 0.018	>100
TKB272 M.W. 653.6936	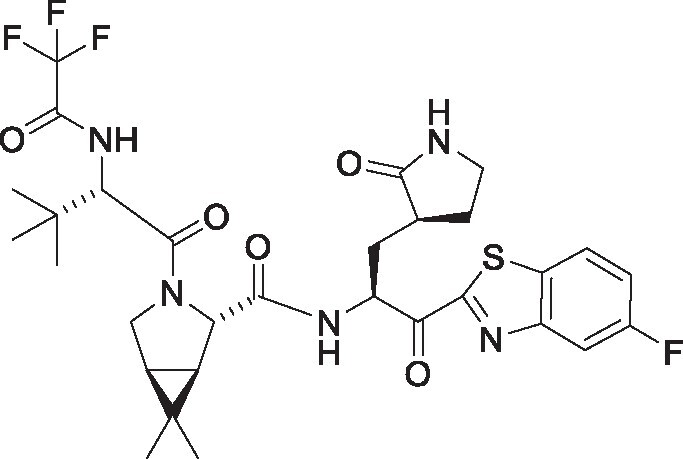	0.0007 ± 0.0004	0.007 ± 0.001	0.0026 ± 0.0010	98 ± 2.1
TKB273 M.W. 653.6936	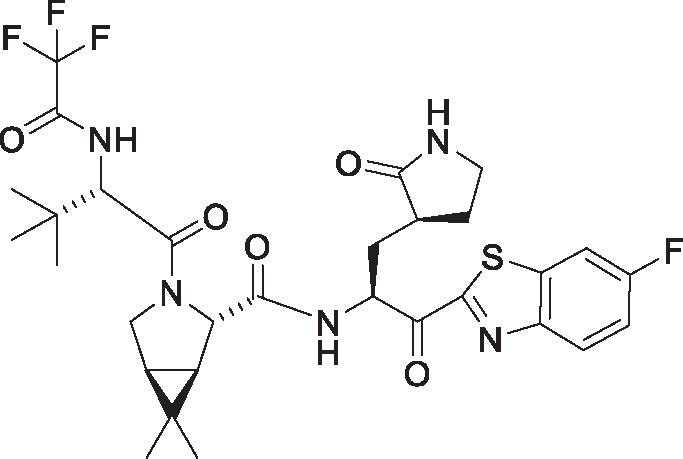	0.0089 ± 0.0041	0.043 ± 0.024	0.114 ± 0.093	>100
TKB252 M.W. 653.6936	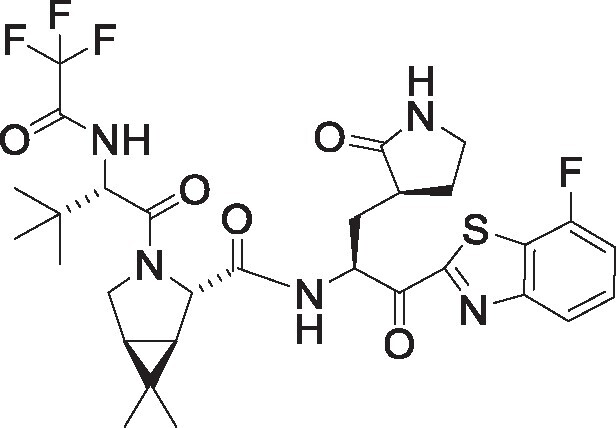	0.20 ± 0.05	0.062 ± 0.026	0.057 ± 0.023	>100
Nirmatrelvir M.W. 499.5352	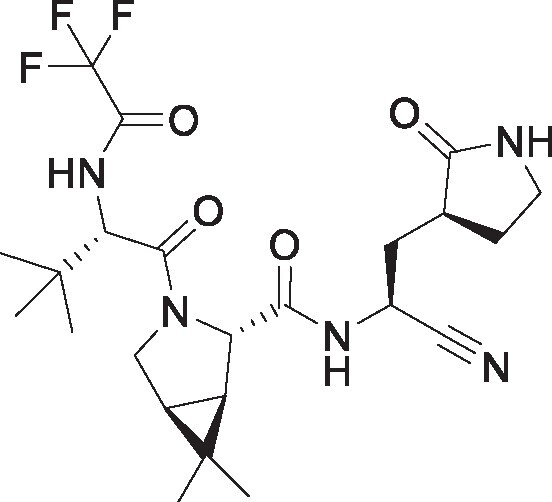	0.0078 ± 0.0013	0.77 ± 0.18	0.051 ± 0.016	>100
Ensitrelvir M.W. 531.88	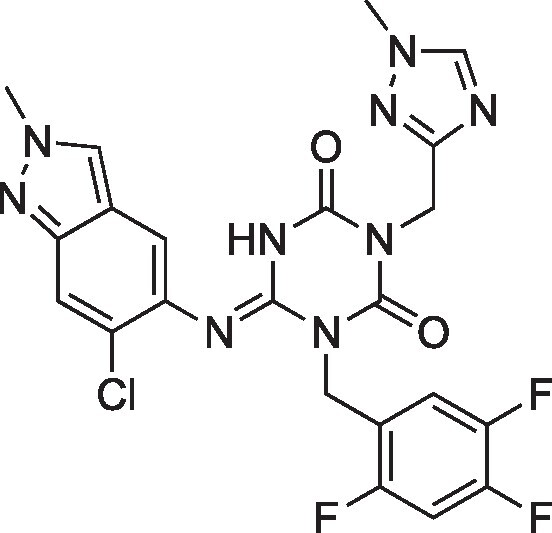	0.0089 ± 0.0006	0.058 ± 0.035	0.051 ± 0.017	>100

Here 50% inhibitory concentration (IC_50_) and 50% effective concentration (EC_50_), 50% cytotoxicity concentration (CC_50_) values were determined as previously published (14). Data from three independent assays are shown as arithmetic means ± 1 SD. Cell-free SARS-CoV-2 M^pro^ enzyme assay was conducted with PRET assays using authentic M^pro^, while cell-based anti-SARS-CoV-2 activity by compounds was determined with RT-qPCR assays of viral RNA from SARS-CoV-2^WK-521^-exposed VeroE6 cells or SARS-CoV-2^BQ.1.1^-exposed HeLa^hACE2-TMPRSS2^ cells. Cytotoxicity of the compounds was evaluated in noninfected VeroE6 cells and was determined with the water-soluble MTT assay.

Since VeroE6 cells are known to abundantly express P-glycoprotein 1 (permeability glycoprotein: P-gp), we determined the anti-SARS-CoV-2 activity in VeroE6 cells in the absence and presence of CP100356, a specific P-gp inhibitor (23). In the presence of CP100356, three compounds, TKB245, TKB272, and nirmatrelvir, were more potent with EC_50_ values of 0.0035, 0.0004, and 0.0363 μM, respectively, showing that CP100356 substantially potentiated their antiviral activity by 9-, 22.5-, and 36.6-fold compared with their activity in the absence of the inhibitor. However, TKB272 is still 9- and 91-fold more potent compared with those of TKB245 and nirmatrelvir in the presence of CP100356 (Table S1).

We next examined antiviral activity of TKB272 vs three selected M^pro^ inhibitors (TKB245, nirmatrelvir, and ensitrelvir) on six various Omicron variants (BA.5, BA.2.75, BQ.1.1, XBB, XBB.1.5, and EG.5.1) in the HeLa^hACE2-TMPPRSS2^ cells-based assay (Table 2). TKB272 exhibited the greatest antiviral activity against all six variants with its EC_50_ values ranging from 0.003 to 0.008 µM, followed by TKB245 with EC_50_ values ranging from 0.006 to 0.038 µM. Nirmatrelvir and ensitrelvir also demonstrated significant activity against all the variants, but their activity was limited (EC_50_ values of nirmatrelvir: 0.02 to 0.75 µM; those of ensitrelvir: 0.06 to 0.22 µM). All inhibition curves for all EC_50_ and CC_50_ values summarized in Tables 1 and 2 were shown in Figs. S3 and S4.

**Table 2.** Effective inhibitory concentrations of M^pro^ inhibitors against a variety of SARS-CoV-2 variants.

	Against SARS-CoV-2 variants EC_50_ (µM)					
	Omicron					
Compound	BA.5	BA.2.75	BQ.1.1	XBB	XBB.1.5	EG.5.1
TKB272	0.008 ± 0.001	0.006 ± 0.003	0.003 ± 0.001	0.003 ± 0.001	0.005 ± 0.002	0.007 ± 0.001
TKB245	0.023 ± 0.009	0.027 ± 0.003	0.038 ± 0.018	0.006 ± 0.003	0.035 ± 0.018	0.009 ± 0.001
Nirmatrelvir	0.75 ± 0.22	0.30 ± 0.22	0.05 ± 0.02	0.02 ± 0.01	0.21 ± 0.28	0.13 ± 0.02
Ensitrelvir	0.20 ± 0.04	0.14 ± 0.06	0.07 ± 0.02	0.06 ± 0.02	0.22 ± 0.09	0.21 ± 0.09

Anti-SARS-CoV-2 activity of compounds discussed in this study was evaluated with RT-qPCR of viral RNA from culture medium of SARS-CoV-2-exposed HeLa^hACE2-TMPRSS2^ cells. Each EC_50_ value was calculated as previously published (14). Data from two to three independent assays are shown as arithmetic means ± 1 SD. Source data are provided as a Source data file.

In terms of human plasma protein binding to small molecule compounds, a1-acid glycoprotein (AAG) is one of the most important binding proteins in plasma, which modulates pharmacokinetics and pharmacodynamics of a variety of drugs. We have previously shown that the presence of high concentrations of AAG significantly reduces the in vitro antiviral activity of HIV-1 protease inhibitors (24). Thus, we examined whether human AAG reduces the activity of nirmatrelvir and TKB272 against SARS-CoV-2. As expected, the presence of 1 mg/mL human AAG (physiological concentrations) significantly reduced the activity of nirmatrelvir (7.6-fold less) and TKB272 (10-fold less) in SARS-CoV-2^XBB1.5^-exposed HeLa^hACE2-TMPRSS2^ cells. Five mg/mL human AAG further reduced the activity of both compounds. It is noteworthy that the EC_50_ value of TKB272 (0.03 µM) in the presence of 1 mg/mL human AAG was much less than that of nirmatrelvir (0.16 µM), suggesting that TKB272 potentially exerts greater activity against SARS-CoV-2 compared with nirmatrelvir (Table S2).

### TKB272 achieves greater or comparable intracellular concentrations compared with nirmatrelvir

**Fig. 1.**
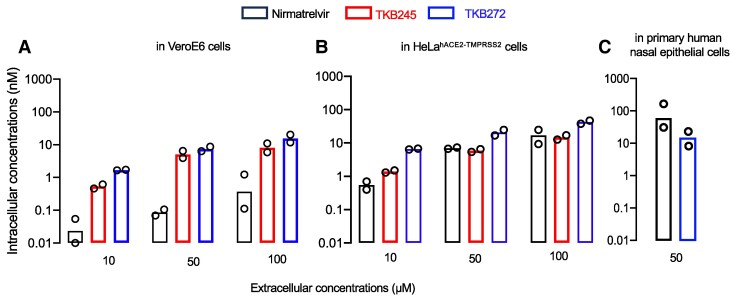
Intracellular concentrations of TKB272. VeroE6 (A) and HeLa^hACE2-TMPRSS2^ cells (B) were incubated with 10, 50, or 100 μM of either nirmatrelvir, TKB245, or TKB272 for 6 h, while primary human nasal epithelial cells were incubated with 50 μM for 24 h (C) Following the incubation, the cells were vigorously washed with PBS, and intracellular concentrations of each compound were determined using LC/MS/MS. Bars indicate geometric means (*n* = 2).

It was noted that while the difference between inhibitory potency of TKB272 against SARS-CoV-2^WK-521^'s M^pro^ (IC_50_ = 0.0007 µM) and that of nirmatrelvir (IC_50_ = 0.0078 µM) was by ∼11-fold, the difference between antiviral activity of TKB272 against SARS-CoV-2^WK-521^ in VeroE6 cells (EC_50_ = 0.007 µM) and that of nirmatrelvir (EC_50_ = 0.77 µM) was greater by 110-fold. The difference in HeLa^hACE2-TMPRSS2^ was by ∼20-fold (0.051 µM/0.0026 µM). We hypothesized that these differences might be attributed to variations in intracellular concentrations of TKB272, TKB245, and nirmatrelvir. When VeroE6 cells were incubated with each agent for 6 h and the intracellular concentrations were quantified using LC/MS/MS, TKB245 and TKB272 concentrations were found higher than those of nirmatrelvir (Fig. 1A). Similarly, intracellular concentrations of TKB272 in HeLa^hACE2-TMPRSS2^ were also greater than those of nirmatrelvir (Fig. 1B). Meanwhile when human primary nasal epithelial cells were incubated with each agent for 24 h and the intracellular concentrations were quantified using LC/MS/MS, TKB272 and these concentrations were found comparable to nirmatrelvir (Fig. 1C). These findings suggest that the varying intracellular concentrations of these compounds may partly contribute to the observed differences in their antiviral activity in this cell-based assay.

### X-ray crystallographic analyses of TKB272 complexed with M^pro^_WT_

**Fig. 2.**
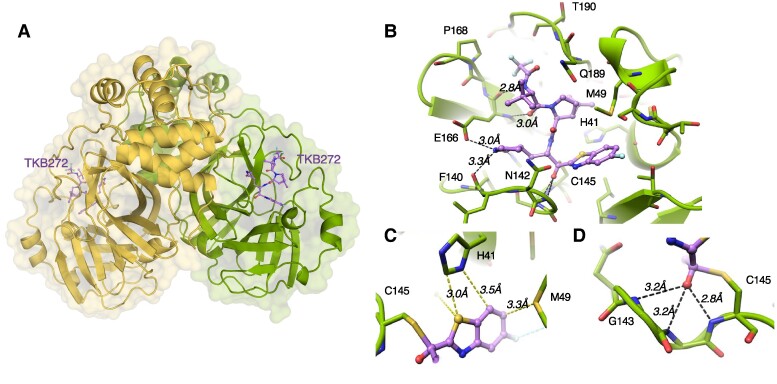
Co-crystal structures of TKB272 with SARS-CoV-2 M^pro^. (A) Surface representation of the homodimer of M^pro^. Protomer A is depicted in yellow, protomer B in green, and TKB272 as purple sticks. (B) Binding mode and hydrogen bond network in M^pro^ complexed with TKB272. Cartoon representation of the crystal structure of M^pro^ is shown in orange complexed with TKB272 (purple sticks). Hydrogen bonds are indicated as black dashed lines. (C) Yellow dashed lines (under 4 Å) indicate van der Waals and sulfur-mediated interactions between the benzothiazole group and surrounding amino acids. (D) The oxyanion hole is formed by three hydrogen bond interactions between the hemiketal oxygen and the backbone amide NH groups of G143, S144, and C145.

**Fig. 3.**
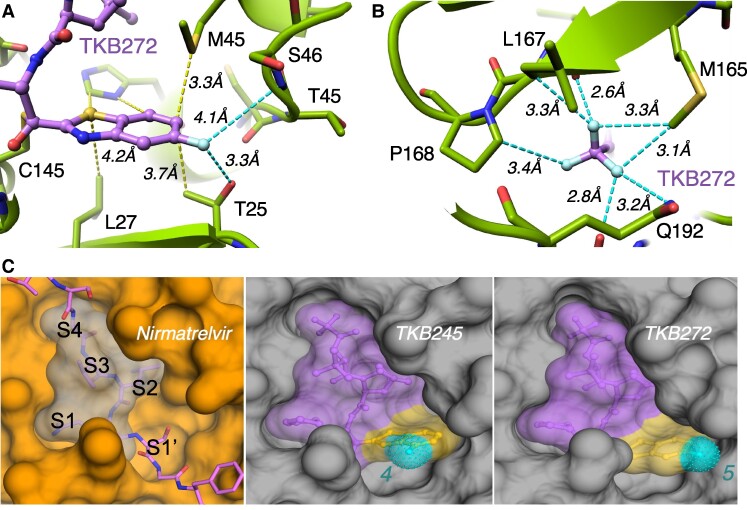
Fluorine-based interactions of TKB272. (A) The fluorine atom located at position 5 within the benzothiazole moiety of TKB272 participates in dual fluorine-based interactions with the side chains of T25 and the main chain nitrogen atom of S46. (B) Furthermore, the trifluoromethyl group forms multiple interactions with the side chains of adjacent amino acids, including M165, L167, P168, and Q192. (C) The surface representation of nirmatrelvir is depicted in gray (left), contrasted with TKB245 and the enhanced TKB272, both of which fill the binding pocket more effectively. The additional benzothiazole moiety is highlighted in yellow, with the fluorine atom at positions 4 and 5 shown in cyan. The original M^pro^_WT_ polyprotein substrate is represented in pink sticks, overlaid by nirmatrelvir (C, left). Both TKB245 and TKB272 filling the binding pocket is more efficient. The subsites (S) in the active-site cavity of M^pro^_WT_ are depicted in the left panel of (C).

TKB272 was built on dimethyl-bicyclo[3.1.0]-proline moiety in the center, γ-lactam as the P1 moiety, and 5-fluorobenzothiazole as the P1′ moiety (Table 1). To gain an insight into the molecular interactions of TKB272 with M^pro^_WT_, we solved an X-ray structure at a resolution of 1.9 Å. In the structures of TKB272, we observed covalent bond formation between C145 and 5-fluorobenzothioazoly ketone (Fig. 2), resulting in a hemithioketal group as in the case of TKB245 (14), the prototype for TKB272. The nitrogen atom of the γ-lactam moiety forms hydrogen bond with the carboxylate side chain of E166 (Fig. 2B) and with the main-chain oxygen of F140 located at a distance of 3.3 Å (Fig. 2B). The 5-fluorobenzothiazole moiety of TKB272 occupied the S1′ subsite, primarily interacting with adjacent residues, particularly M49, as well as the catalytic residue H41 (Fig. 2C). TKB272 establishes eight direct hydrogen bonds with the M^pro^_WT_ catalytic site, including three with the backbone NH of G143, S144, and C145 in the oxyanion hole (Fig. 2D). In addition, we observed the significant role of a sulfur-based interaction between benzothiazole and M49. It is important to highlight that nirmatrelvir lacks a P1′ moiety, leaving the S1′ subsite empty (Fig. 3C).

### Structural analysis of TKB272's fluorine-associated interactions with M^pro^

To gain a detailed insight into the 5-fluorine atom-associated molecular interactions of TKB272 with M^pro^, we solved the X-ray structure of the complex at a resolution of 2.2 Å. In our analysis, we uncovered intriguing fluorine-based interactions involving TKB272, as illustrated in Fig. 3. Specifically, the fluorine atom added at the 5th position in the benzothiazole moiety of TKB272 was observed to engage in two distinctive interactions. Firstly, it established fluorine-based interactions with the side chains of T25 and the main chain nitrogen atom of S46, implying a significant role in stabilizing the TKB272's binding within the active site. Notably, this role was absent in the previously published compound TKB245, where the fluorine at the 4th position was exposed to the solvent, lacking any significant interactions with binding pocket residues (14).

Additionally, we found critical interactions occurring around the trifluoromethyl group of TKB272, forming a complex network of interactions with the side chains of neighboring amino acids, including M165, L167, P168, and Q192. However, it is worth noting that these interactions are also conserved in the nirmatrelvir complex structure (4). Therefore, we posit that these interactions alone would not be the reason for the superior potency of TKB272 over nirmatrelvir or TKB245 in terms of the compound's overall binding affinity. Instead, it is likely that the 5-fluorobenzothiazole moiety of TKB272, which effectively fills the binding pocket and forms additional interactions (Fig. 3C), plays a pivotal role in enhancing its binding affinity. Collectively, these structural insights strongly suggest that the 5-fluorobenzothiazole moiety plays a crucial role in the potent activity of TKB272 against SARS-CoV-2 strains compared with the activity of nirmatrelvir.

### Orally administered TKB272 potently blocks the infectivity and replication of SARS-CoV-2^XBB.1.5^ in mice without ritonavir

We next attempted to examine whether TKB272 blocked the infectivity and replication of a SARS-CoV-2 variant, SARS-CoV-2^XBB.1.5^, in XBB.1.5-susceptible B6.Cg-Tg(K18-hACE2)2-Prlmn/J-transgenic mice. Prior to the experiment, we performed a typical PK assay of TKB272 in C57BL/6JJcl mice (*n* = 3) using three different doses of TKB272 (50, 80, and 100 mg) (Fig. S5) and also determined PK of TKB272 in human liver-chimeric mice, PXB mice, which are thought to be of utility in well-addressing early safety assessment and characterization of drug metabolism close to those in humans (14, 25). As perorally administered to the mice, the half-life of TKB272 (3.12 h in Fig. S6a) was longer that of nirmatrelvir (1.43 h in Fig. S6a). As orally administered in ICR mice (Fig. S6b), the half-life of TKB272 (3.37 h in Fig. S6c) was similar to that seen in PXB-mice (Fig. S6a).

**Fig. 4.**
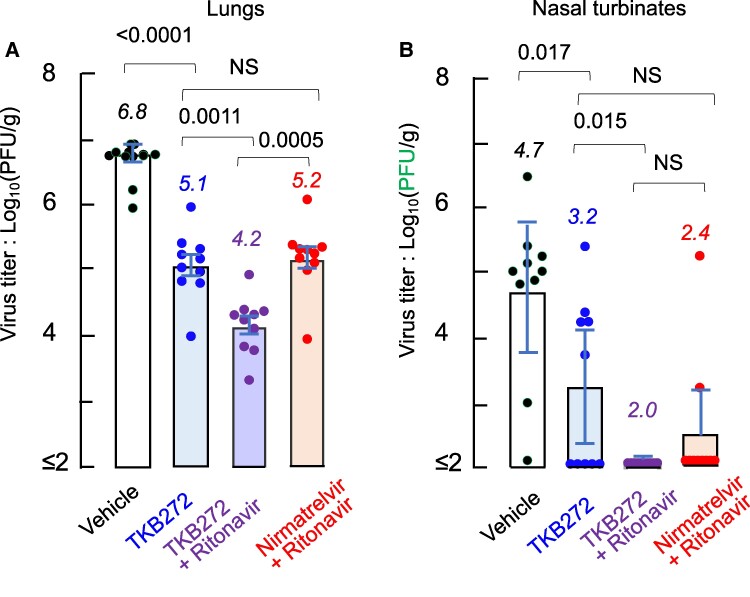
In vivo efficacy of TKB272 against SARS-CoV-2^XBB1.5^-infected B6.Cg-Tg(K18-hACE2)2 Prlmn/J transgenic mice. Ten K18-hACE2 mice per group were challenged with SARS-CoV-2^XBB1.5^ (1 × 10^4^ PFU). Two hours later, animals were treated with: 50 mg/kg TKB272, 50 mg/kg TKB272 + 3 mg/kg ritonavir, 50 mg/kg nirlatrelvir + 3 mg/kg ritonavir or vehicle (placebo) orally twice daily for 2 days. Animals were euthanized on 2-days post infection and, lungs (A) and nasal turbinates’ (B) were collected for determination of virus titers using VeroE6^TMPRSS2^ cells. Bars indicate mean values and error bars represent standard deviations. All *P*-values were calculated using the Wilcoxon's signed rank-sum test with two-sided, and no multiple adjustment was made.

In an attempt to determine the in vivo efficacy of TKB272, 10 K18-hACE2 mice were exposed to SARS-CoV-2^XBB1.5^ (1 × 10^4^ PFU) and the animals were orally administered with (i) 50 mg/kg TKB272, (ii) 50 mg/kg TKB272 + 3 mg/kg ritonavir, (iii) 50 mg/kg nirmatrelvir + 3 mg/kg ritonavir, or (iv) vehicle twice daily for 2 days. The animals were then euthanized on 2-days post-infection, and lungs and nasal turbinates were collected for determination of virus titers using VeroE6^TMPRSS2^ cells (14). In the lung from the mice receiving only a vehicle, as high as 10^6.8^ PFU/g viral titer was seen, whereas the tissue from mice receiving TKB272 without ritonavir gave a significantly less titer with 10^5.1^ PFU/g (*P* < 0.0011). Nirmatrelvir with ritonavir gave comparably lower viral titer (10^5.2^ PFU). With ritonavir, TKB272 further reduced the titer with 10^4.2^ (*P* = 0.0011) (Fig. 4A). The viral titers determined in nasal turbinates (Fig. 4B) were generally lower than in lung tissue, but the general viral titer profiles are similar to those in lung tissue. The data together strongly suggest that even with the absence of ritonavir, TKB272 exerts as potent anti-SARS-CoV-2 activity as nirmatrelvir with ritonavir, which should mitigate the adverse effects of the therapy with nirmatrelvir plus ritonavir.

### Nirmatrelvir lost its activity against SARS-CoV-2^E166V-P14^ by 71-fold (EC_50_ = 1.42 µM), while TKB272 by 17.7-fold (EC_50_ = 0.0513 µM)

**Fig. 5.**
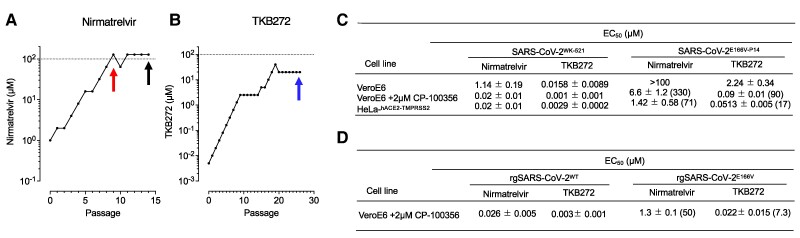
Nirmatrelvir lost its activity against SARS-CoV-2^E166V-P14^ by 71-fold (EC_50_ = 1.42 µM), while TKB272 by 17.7-fold (EC_50_ = 0.0513 µM). SARS-CoV-2^WK521^ was propagated in VeroE6^TMPRSS2^ cells in the presence of increasing concentrations of nirmatrelvir a or TKB272 b. SARS-CoV-2^WK-521^ was first seen to have acquired an E166V substitution in the M^pro^-encoding gene at passage 9 (highlighted in a red arrow in Panel A). SARS-CoV-2WK-521 vigorously replicated in the presence of nirmatrelvir at as high as 128 µM (passages 11 to 14). As selected with TKB272, the virus stopped replicating in the presence of 20 µM TKB272 and no significant amino acid substitution was found until passage 25 (highlighted in a blue arrow in Panel (B)). The E166V-containing virus obtained at passage 14 with nirmatrelvir (SARS-CoV-2^E166V-P14^ highlighted in a black arrow in Panel (A) was used in the cell-based antiviral assay, where SARS-CoV-2^E166V-P14^ proved to be highly resistant to nirmatrelvir but was found sensitive to TKB272 (C). More than 99.8% of the SARS-CoV-2^E166V-P14^ population had only E166V substitution as assessed with deep sequencing. SARS-CoV-2^WK-521^ propagated in the presence of as high as 20 µM TKB272 contained no amino acid substitutions. A recombinant SARS-CoV-2 variant carrying E166V substitution alone (rgSARS-CoV-2^E166V^) and a recombinant wild-type virus (rgSARS-CoV-2^WT^) were generated through the reverse genetics technology. The susceptibility of both strains was determined against nirmatrelvir and TKB272. Values shown in parentheses are fold-changes in IC_50_ values relative to the values for rgSARS-CoV-2^WT^ (D). Data in Panels (C) and (D) are from three independent assays and show arithmetic means (µM) ±1 SD.

SARS-CoV-2 variants reportedly phenotypically resistant to the first-generation drugs might have been naturally occurring (26) or might emerge because of the fast replication speed of SARS-CoV-2. Although such variants might emerge in part because of the limited potency of such drugs as in the case of anti-HIV drugs (27, 28), the SARS-CoV-2 resistance issues require further research. Thus, we attempted to generate SARS-CoV-2 resistant to nirmatrelvir and TKB272 by propagating SARS-CoV-2^WK-521^ in the presence of increasing concentrations nirmatrelvir or TKB272 in VeroE6^TMPRSS2^ cells. When SARS-CoV-2^WK-521^ was propagated in the presence of nirmatrelvir, the virus was first to seen to acquire E166V substitution in the M^pro^-encoding gene of the virus at early as passage 9, however, when the same virus was selected with up to 20 µM TKB272, no significant amino acid substitution was identified until passage 25 (Fig. 5A and B). The nirmatrelvir-selected virus obtained at passage 14 (SARS-CoV-2^E166V-P14^) proved to contain E166 V by more than 99.8% as assessed with deep sequencing of ∼4,000 sequences of the M^pro^-encoding gene and no other substitutions were identified (Fig. S7).

We then examined the susceptibility of the wild-type SARS-CoV-2^WK-521^ and SARS-CoV-2^E166V-P14^ against nirmatrelvir and TKB272. The EC_50_ values of nirmatrelvir and TKB272 against SARS-CoV-2^WT^ were 0.02 and 0.0029 µM in HeLa^hACE2-TMPRSS2^ cells, which however went up to 1.42 and 0.0513 µM against SARS-CoV-2^E166V-P14^ (Fig. 5C). If we compare the fold-difference, the potency of nirmatrelvir went low by 71-fold, while that of TKB272 by17.7-fold. We interpreted the difference between 71-fold and 17.7-fold substantial. It is also noteworthy that the absolute EC_50_ values of nirmatrelvir and TKB272 against SARS-CoV-2^E166V-P14^ in HeLa^hACE2-TMPRSS2^ cells were 1.42 and 0.051 µM. With the E166 V substitution, TKB272 lost its activity by 17.7-fold, while nirmatrelvir lost its activity against SARS-CoV-2^E166V^ by 71-fold. These data suggest that the E166 V substitution confers less loss of activity to TKB272. We further addressed the issue of cross-resistance of antiviral drugs as reported by Iketani et al. (29), and determined that the EC_50_ values of molnupiravir (nucleoside inhibitor) against SARS-CoV-2^E166V-P14^ (0.75 µM) and SARS-CoV-2^WK-521^ (0.84 µM) were comparable, while the EC_50_ values of ensitrelvir (M^pro^ inhibitor) against SARS-CoV-2^E166V-P14^ (0.54 µM) and SARS-CoV-2^WK-521^ (0.03 µM) showed a 18-fold difference (Table S3). These data, taken together, strongly suggest that although TKB272 has certain cross-resistance to M^pro^ inhibitors as observed in ensitrelvir, TKB272 remains largely effective against SARS-CoV-2^E166V-P14^.

We also newly generated a SARS-CoV-2 variant carrying only E166V substitution (rgSARS-CoV-2^E166V^) and its background wild-type virus (rgSARS-CoV-2^WT^) through the reverse genetics technology and have evaluated the sensitivity of a recombinant SARS-CoV-2 to antiviral compounds. The EC_50_ values of nirmatrelvir and TKB272 against rgSARS-CoV-2^WT^ were 0.03 and 0.003 µM in VeroE6 cells with 2 µM CP-100356, which however went up to 1.3 and 0.022 µM against rgSARS-CoV-2^E166V^, respectively. When we compare the fold-difference, the potency of nirmatrelvir went low by 50-fold, while that of TKB272 by 7.3-fold (Fig. 5D). Although molnupiravir showed similar EC_50_ values against both viruses tested, EC_50_ value of ensitrelvir against rgSARS-CoV-2^E166V^ was moderately reduced compared with that of rgSARS-CoV-2^WT^ and the potency of ensitrelvir went low by 12-fold (Table S3).

These data, taken together, strongly suggest that although TKB272 has certain cross-resistance to M^pro^ inhibitors as observed in ensitrelvir, TKB272 remains largely effective against SARS-CoV-2^E166V-P14^ and rgSARS-CoV-2^E166V^.

### TKB272 and nirmatrelvir bind to M^pro^_WT_ and promote its dimerization, while E166V substantially reduces nirmatrelvir's binding to M^pro^_E166V_ and its dimerization

**Fig. 6.**
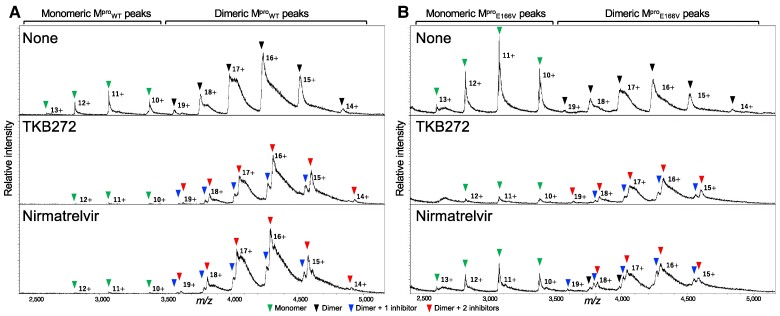
Native mass spectrometric analysis of M^pro^_WT_ and M^pro^_E166V_ treated with TKB272 or nirmatrelvir. 7.5 µM of M^pro^_WT_ (A) or M^pro^_E166V_ (B) was treated with 15 µM of each M^pro^ inhibitor and subjected to native mass spectrometric analysis. Relative mass spectra of each M^pro^ with or without TKB272 bound or nirmatrelvir bound are shown. Charge states 10+, 11+, 12+, and 13+ are annotated to mass spectra corresponding to monomeric M^pro^ species and charge states 14+, 15+, 16+, 17+, 18+, and 19+ are annotated to mass spectra corresponding to dimeric M^pro^ species. The peaks are annotated with the green triangles for the monomer, black for the dimer, blue for the dimer with one inhibitor, and red for the dimer with two inhibitors.

Since SARS-CoV-2^E166V-P14^ proved highly resistant to nirmatrelvir while the activity of TKB272 against SARS-CoV-2^E166V-P14^ was not that much affected by the substitution, the modes of the binding of nirmatrelvir and TKB272 to M^pro^_WT_ and M^pro^_E166V_ was examined using native mass spectrometry. In the chart of relative mass spectra of M^pro^ without inhibitor shown in the top panel of Fig. 6A, two types of major peaks, monomeric and dimeric ones, were seen. When we calculated the peak ratios of the authentic M^pro^_WT_ monomers and dimers, the enzyme exhibited an equilibrium of a monomer percentage of 22.6% and a dimer percentage of 77.4% under the measurement conditions (top panel of Fig. 6A and Fig. S8a). Upon treatment with TKB272 and nirmatrelvir (in the middle and bottom panels of Fig. 6A, respectively), the equilibrium shifted significantly toward dimeric M^pro^_WT_-inhibitor complexes, with no substantial differences observed between the two inhibitors (in the middle and bottom panels of Fig. 6A and Fig. S8b and c).

In contrast, M^pro^_E166V_ showed a substantial shift to a monomer-dominant equilibrium with a monomer percentage of 61.3% and a dimer percentage of 38.7% (top of Fig. 6B and Fig. S8d), suggesting that E166 affects the initiation of enzyme dimerization and that the proportion forming M^pro^_E166V_ dimers is much less than that of M^pro^_WT_. However, treating M^pro^_E166V_ with TKB272 resulted in a pronounced shift in equilibrium toward dimeric M^pro^_E166V_-inhibitor, suggesting that TKB272 fairly well binds to M^pro^_E166V_, causing dimerization (middle of Fig. 6B). Of note, when M^pro^_E166V_ was treated with nirmatrelvir, the monomer peaks of 10^+^, 11^+^, 12^+^, and 13^+^ charge states were substantially greater than those in dimeric M^pro^_E166V_-TKB272 (bottom of Fig. 6B and Fig. S8), suggesting that the binding of nirmatrelvir to M^pro^_E166V_ became weak.

### Structural insight of TKB272's persisting potency against SARS-CoV-2^E166V-P14^

**Fig. 7.**
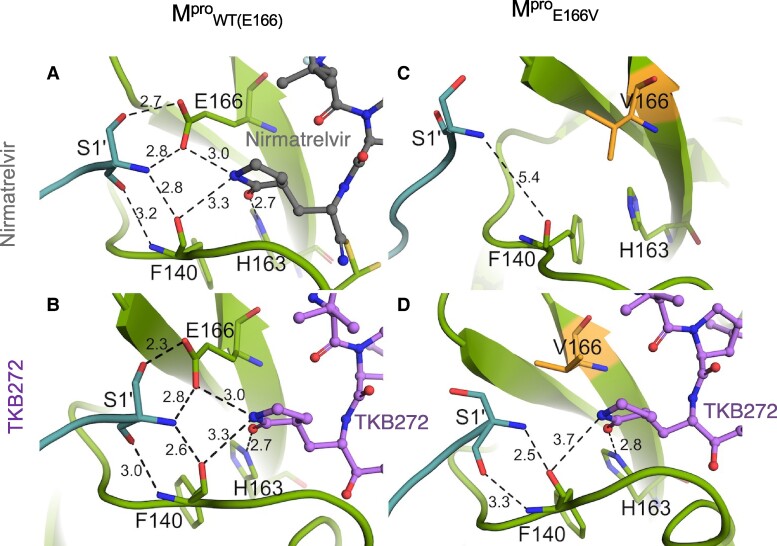
M^pro^_E166V_ fails to complex with nirmatrelvir, but readily complexes with TKB272. S1′ residues from the second protomer of M^pro^_WT(E166)_ exhibit multiple hydrogen bond interactions with both the carboxyl group of E166 and the main chain of F140, with similar hydrogen bond patterns observed for both nirmatrelvir (gray) (A) and TKB272 (purple) (B). In the presence of the E166V substitution, nirmatrelvir is no longer detected within the binding pocket, causing S1′ to disengage, resulting in a distance of 5.4 Å between them (C). TKB272 remains co-crystallized with MproE166V, albeit with a loss of contact between its g-lactam moiety and the side chain of E166. Nevertheless, S1′ continues to establish two hydrogen bonds with F140 (2.5 Å and 3.3 Å) (D).

As TKB272 remained potent against SARS-CoV-2^E166V-P14^ while nirmatrelvir lost its activity, and E166V reduces nirmatrelvir-M^pro^ binding and dimerization as described above, we extended our efforts to obtain the insight into the structural changes that occur with the E166V substitution. In the authentic M^pro^_WT(E166)_ complexed with nirmatrelvir (Fig. 7A) or TKB272 (Fig. 7B), it is noted that the oxygen of the γ-lactam moiety forms a strong hydrogen bond with the side chain of His-163 (2.7 Å) (Fig. 7A and B). In addition, the nitrogen atom of the γ-lactam moiety forms hydrogen bond with the carboxylate side chain of Glu-166 (3.0 Å) (Fig. 7A and B) and with the main-chain oxygen of F140 located at a distance of 3.3 Å (Fig. 7A and B). It is of note that S1′ residues from the second protomer forms four strong hydrogen bonds with both the carboxyl group of E166 and the main chain of F140 (Fig. 7A and B). However, the X-ray structure of M^pro^_E166V_ with nirmatrelvir yielded an apo structure devoid of any nirmatrelvir density. This absence of density can be attributed to the reduced affinity of nirmatrelvir for the E166V variants (Fig. 7C). In this apo structure (Fig. 7C), we observed a significant shift in the position of the N-terminal residue S1′. The substitution of the acidic side chain of glutamate with the hydrophobic residue valine appears to be the primary reason for this reduction in binding affinity toward nirmatrelvir. On the contrary, we successfully obtained the complex of TKB272 with M^pro^_E166V_ (Fig. 7D), and we noted that most of the interactions, including those involving S1′, remain largely intact, albeit with minor changes (Fig. 7D).

### TKB272 shows no apparent genotoxicity as tested in the micro-Ames test

To further test the safety of the TKB272 for possible further drug development, we performed “µAmes” bacterial mutation test. TKB272 did not inhibit the growth of bacterial strains employed, regardless of the presence or absence of metabolic activation. TKB272 did not increase the number of revertant colonies more than twice compared with the negative control value in any strains, regardless of metabolic activation (Table S4).

## Discussion

An M^pro^ inhibitor, nirmatrelavir, has been clinically available for the treatment of mild-to-moderate COVID-19 under the emergency use authorization by FDA, and another M^pro^ inhibitor, ensitrelvir has also been approved for emergency use in mild COVID-19 patients in Japan. If the EC_50_ values of the currently available anti-COVID-19 agents are determined in test tubes, the order of antiviral potency against various SARS-CoV-2 strains would appear to be in large nirmatrelvir ≈ ensitrelvir > molnupiravir > remdesivir (13, 14, 28), although the potency order varies depending on the method, strains and cells used. Moreover, nirmatrelavir has to be administered with CYP3A4-inhibiting ritonavir, and is contraindicated with a number of drugs dependent on CYP3A4 for metabolism, otherwise resulting in serious and/or life-threatening reactions (30). Ensitrelvir is not co-administered with ritonavir but is by itself a strong inhibitor of CYP3A4/5 and is contraindicated with various drugs (31). Ensitrelvir is also contraindicated in pregnant or possibly pregnant individuals because of potential fetal harm documented in animals. Thus, for more effective response to the present COVID-19 pandemic, more potent and more tolerable antiviral therapeutics are urgently required.

Fluorination often increases metabolic stability, delays inactivation of drugs, and elongates dosage periods because C–F bond is highly stable (16, 17, 24, 25, 27, 32, 33). Fluorination also increases lipophilicity due to its greater hydrophobicity than C–H bond, often increasing cell membrane penetration. Thus, in the present study, based on our observation on the previously published coronavirus M^pro^ inhibitors (13, 14, 21, 34–36), we designed, synthesized, and identified TKB272, using fluorine-scanning of a benzothiazole, a critical moiety as M^pro^ inhibitors (Table 1). Notably, TKB, as orally administered, is so potent and is capable of suppressing SARS-CoV-2 without using ritonavir as effectively as nirmatrelvir does with ritonavir in mice (Fig. 4). If administered with RTV, TKB272 exerted far more potent anti-SARS-CoV-2 activity (Fig. 4).

The present native mass spectrometric analysis revealed that the addition of TKB272 as well as nirmatrelvir substantially shifted the equilibrium toward a greater abundance of dimers, predominantly bound by two inhibitor molecules, suggesting that these two inhibitors inhibit the enzymatic activity in a manner in which these inhibitors expedite dimerization and stabilize the dimerization of monomers to the extent so that M^pro^ inhibitors occupy the enzymatic active site hydrophobic cavity highly effectively and prevent the entry of SARS-CoV-2 immature polyproteins, which are otherwise processed by Mpro (Fig. 6). The promoted protease protomer dimerization triggered by inhibitors has also been seen in the interactions of HIV-1 protease and its inhibitors, in which in the presence of HIV-1 protease inhibitors (such as saquinavir and darunavir) the amounts of HIV-1 protease monomers are reduced, forming dimers bound to the inhibitors (37).

Structurally, nirmatrelvir lacks any P1′ moiety, which leaves the S1'subsite empty (left, Fig. 3C), however, TKB272 has the P1′-well-filling benzothiazole moiety (right, Fig. 3C) as well as a fluorine atom at 5-position of the benzothiazole that forms effective halogen bonds with amino acids of the active-site cavity of M^pro^_WT_ (Fig. 3A), playing a pivotal role in enhancing TKB272's binding affinity to M^pro^_WT_. Regarding the role of E166, similar to our findings shown in Figures 2 and 6, previous studies by Duan et al (38) focusing on the molecular mechanisms of SARS-CoV-2 resistance to nirmatrelvir and ensitrelvir show that the E166V substitution results in a loss of one hydrogen bond with nirmatrelvir. However, this substitution did not affect the affinity of ensitrelvir. This is because ensitrelvir does not directly form a hydrogen bond with the side chain of E166 and other hydrogen bonds between M^pro^_WT_ and ensitrelvir remained unaffected. This underscores the critical role of the E166 side chain contact with NH group of the lactam ring of nirmatrelvir (Fig. 7A). Despite the structural similarity of TKB272's chemical scaffold to that of nirmatrelvir, the presence of an additional 5-fluorobenzothiazole ring substantially contributes to TKB272's robust affinity toward the SARS-CoV-2 Mpro (Fig. 3C). It is noteworthy that this remarkable binding affinity of TKB272 persists even in the presence of the E166V substitution as seen in the TKB272-M^pro^_E166V_ complex (Fig. 7D).

Regardless of the mechanism(s), the results reported here demonstrate that the addition of a simple 5-fluorinated benzothiazole can convert nirmatrelvir that has no fluorine atom or benzothiazole moiety to TKB272, a compound with the capacity to inhibit the replication of SARS-CoV-2 in vivo highly potently without ritonavir. As for the possible safety of TKB272, we have performed the micro-Ames test, which showed no genotoxicity; however, careful tests including potential fetal harm and such have to be conducted upon further clinical development of TKB272. We should also stress that the present data do not indicate that TKB272 is superior to nirmatrelvir in a possible clinical setting. Only double-blind, properly controlled clinical trials comparing the efficacy of nirmatrelvir and that of TKB272/TKB245 can teach us whether TKB272/TKB245 is worth adding to the armamentarium in our combat with COVID-19. It is to be added as well that the findings in the present in vitro study using cell lines and a few virus strains does not give us an appropriate prediction of the antiviral efficacy of test compounds. At the present time when widespread vaccination was accomplished, herd immunity gained, and the virulence of the virus relatively weakened, highly potent antiviral agents may not immediately be developed by pharmaceutical firms. However, results of detailed studies of antiviral agents such as TKB272 should be critical for our preparation for the arrival of next potential pandemic threats. The present results may have implications in the development of new strategies for the pharmacologic interventions against the pathogenic coronaviruses including SARS-CoV-2, although more careful studies have to be conducted. Furthermore, highly potent TKB272 may serve as a COVID-19 therapeutic, overcome resistance to existing M^pro^ inhibitors, and might pave the way to prevent long COVID if long COVID proves to be related to limited antiviral activity of the present therapy.

## Materials and methods

### Cells and viruses

VeroE6 cells were obtained from the American Type Culture Collection (ATCC) (CRL-1586) (Manassas, VA) and were maintained in Dulbecco's modified Eagle's medium (d-MEM) supplemented with 10% fetal bovine serum (FCS), 100 μg/mL of penicillin, and 100 μg/mL of streptomycin. VeroE6^TMPRSS2^ and HeLa^hACE2-TMPRSS2^ cells were obtained from the Japanese Collection of Research Bioresources (JCRB) Cell Bank (JCRB1835, Osaka, Japan) and were maintained the same conditioned medium as that VeroE6 cell line except for G418 (0.5 mg/mL) addition. HEK293T cells were cultured in DMEM supplemented with 10% FCS. All cells were maintained at 37°C with 5% CO_2_. The cells were regularly tested and confirmed to be negative for mycoplasma contamination by using PCR. SARS-CoV-2 strain JPN/TY/WK-521/2020 (SARS-CoV-2^WK-521^) was obtained from the National Institute of Infectious Diseases (Tokyo, Japan). Six clinically isolated SARS-CoV-2 mutant strains were used in the current study: a BA.2.75 (omicron BA.2.75) strain (hCoV-19/Japan/TY41-716/2022 [SARS-CoV-2^TY41–716^, GISAID Accession ID; EPI_ISL_13969765]) and a XBB.1.5 (omicron XBB.1.5) strain (hCoV-19/Japan/23-018-P1/2022 [SARS-CoV-2^23–018-P1^, GISAID Accession ID; EPI_ISL_16889601]) were obtained from National Institute of Infectious Diseases, Tokyo, Japan. A BA.5 (omicron BA.5) strain (hCoV-19/Japan/TKYTS14631/2022 [SARS-CoV-2^TKYTS14631^, GISAID Accession ID; EPI_ISL_12812500.1]), a XBB (omicron XBB) strain (hCoV-19/Japan/TY41-795/2022 [SARS-CoV-2^TY41–795^, GISAID Accession ID; EPI_ISL_15669344]), a BQ.1.1 (omicron BQ.1.1) strain (hCoV-19/Japan/TY41-796/2022 [SARS-CoV-2TY41-796, GISAID Accession ID; EPI_ISL_15579783]), and a EG.5.1 (omicron EG.5.1) strain (hCoV-19/Japan/TKYnat14564/2023 [SARS-CoV-2^TKYnat14564^, GISAID Accession ID; EPI_ISL_18082364]) were provided from Tokyo Metropolitan Institute of public Health, Tokyo, Japan. An XBB.1.5 (omicron XBB.1.5) strain (hCoV-19/USA/MD-HP40900-PIDYSWHNUB/2022 [SARS-CoV-2^MD-HP40900-PIDYSWHNUB^, GISAID Accession ID; EPI_ISL_16026423]) was obtained from the University of Tokyo, Tokyo, Japan (39). All variants used in this study were listed in Table S5.

### Antiviral agents

TKB245, TKB272, TKB273, and TKB252 are all small molecule inhibitors of main protease of SARS-CoV-2 (M^pro^) containing benzothiazole moiety and were designed and synthesized in the present study, and their purity was >95% or 99% as assessed with high-performance liquid chromatography (HPLC). M^pro^ inhibitors containing 4-fluorobenzothiazole-2-carbonyl moieties such as TKB245 have been recently reported (14, 40, 41). Chemical structures of all the antiviral agents are illustrated in Table 1. Detailed synthetic methods for all TKB compounds except TKB245 will be described elsewhere by H. Tamamura et al. nirmatrelvir (4) (#HY-138687, purity: >99.93%, MedChemExpress), ensitrelvir (15) (#HY-143216A, purity: >98%, MedChemExpress), and ritonavir (#R0116, purity: >98.0%, Tokyo Chemical Industry) were purchased. Each compound was dissolved in DMSO at 20 mM as stock solutions.

### Antibodies used and validation

For immunocytostaining, COVID-19 convalescent plasma-derived lgG (ConvlgG) was used as a primary antibody (1/500 dilution) (lgG was purified at National Center for Global Health and Medicine), while Alexa Fluor 488 AffiniPure Fab Fragment Goat Anti-Human lgG (H + L) was used for a secondary antibody (1/200 dilution) (Jackson lmmunoResearch, 109-547-003). SARS-CoV-2 infection and lgG amounts were determined with RNA-qPCR and ELISA, respectively. ConvlgG was validated using immunostaining of SARS-CoV-2-infected and -uninfected VeroE6 cells and the data obtained were confirmed to be free from nonspecific detection.

### SARS-CoV-2 M^pro^ and human cysteine protease enzyme assay

The 3CLpro (SARS-CoV-2) assay kits (BPS Bioscience, San Diego CA, cat. nos. 78042-2 and 78350-1) are designed to measure 3CLpro activity and identify inhibitors of this enzyme, while human cathepsin L inhibitor screening kit (abcam, cat. Cambridge, UK, no. ab197012) and human calpain activity assay kit (AnaSpec. Inc. Fremont CA, cat. no. AS-72149) are designed to measure human cysteine protease activity and identify inhibitors of these enzymes. These assays were performed in a 96-well plate using a fluorogenic substrate. Briefly, a solution of each enzyme was prepared according to the manufacturer's protocol in assay buffer. Separately, solutions of test compounds necessary to generate a seven-point dose response curve were prepared in half-log serial dilution. Test compounds were added to the plate, and the mixture was preincubated for 30 min at room temperature. A blank well (no enzyme) was included to assess the background signal, while the known inhibitors GC376, FF-FMK and B27-WT were used as positive controls for SARS-CoV-2 M^pro^/3CLpro, cathepsin L, and calpain, respectively. The plates of SARS-CoV-2 M^pro^, cathepsin L, and calpain were incubated with each fluorogenic substrate for 4, 0.3, and 1 h, respectively, at room temperature. Then fluorescence intensity was measured in a Cytation 5-cell imaging multimode reader (BioTek, Winooski, VT, USA) (excitation/emission: 360/460 nm, excitation/emission: 400/505 nm, and excitation/emission: 490/520 nm), respectively. End point fluorescence intensities were measured, the blank values were subtracted from all values, and only the linear part of the curves was used for the evaluations.

### Antiviral activity and cytotoxicity assays

For general cell-based antiviral assay, VeroE6 or HeLa^hACE2-TMPRSS2^ cells were seeded in a 96-well plate (2 × 10^4^ cells/well) and incubated for 1 day, then the virus was inoculated into the culture at each multiplicity of infection (MOI): SARS-CoV-2^WK-521^, 80; SARS-CoV-2^TY41–716^ (omicron BA.2.75), 40; SARS-CoV-2 ^23–018-P1^ (omicron XBB.1.5), 100; SARS-CoV-2^14631^ (omicron BA.5), 30; SARS-CoV-2^TY41–795^ (omicron XBB), 100; SARS-CoV-2^TY41–796^ (omicron BQ.1.1), 80; SARS-CoV-2^TKYnat14564^ (omicron EG.5.1), 100; SARS-CoV-2M^pro^_E166V_ (selected nirmatrelvir-resistant variant), 80. The MOI values were determined by dividing the viral copy number (quantified with RT-qPCR) by the number of plated targeted cells. One hour post exposure, the virus was removed and the cells were washed out one time with culture medium and incubated for 3–4 days with each drug solution. Following the incubation, culture supernatants were harvested and viral RNA was extracted using a QIAamp viral RNA minikit (Qiagen, Hilden, Germany), and RT-qPCR was then performed using One Step PrimeScript III RT-qPCR mix (TaKaRa Bio, Shiga, Japan) and a 7500 Fast Real-Time PCR Instrument (Applied Biosystems, Waltham, MA, USA) following the instructions of the manufacturers. The primers and probe used for detecting SARS-CoV-2 nucleocapsid (1) were 5′-AAATTTTGGGGAC-CAGGAAC-3′ (forward), 5′-TGGCAGCTGTGTAGGTCAAC-3′ (reverse), and 5′-FAM-ATGTCGCGCATTGGCATGGA-black hole quencher 1 (BHQ1)-3′ (probe).

Meanwhile, the effect of the presence of P-glycoprotein inhibitor, CP-100356 (efflux inhibitor, EI) (Sigma-Aldrich, Co. LLC), on the antiviral activity of nirmatrelvir and TKB272 was examined using SARS-CoV-2^WK-521^-exposed VeroE6 cells. The effect of the presence of Human aipha-1-acid glycoprotein (hAAG) (FUJIFILM Wako Pure Chemical Co. Osaka, Japan), which is one of human plasma proteins and functions as a carrier for basic or neutral lipophilic compounds, on the antiviral activity of nirmatrelvir and TKB272 was also evaluated using SARS-CoV2^XBB1.5^-exposed HeLa^hACE2-TMPRSS2^ cells. In the experiments using primary human airway epithelial cells, human nasal epithelial cells HNEpC (#C-12620; Promo Cell, Heidelberg Germany) were cultured in an airway epithelial cell growth medium (C-21160; PromoCell, Heidelberg, Germany) and supplement (C-39165; PromoCell, Heidelberg, Germany), were seeded in a 96-well plate (2 × 10^4^ cells/well), and incubated for 1 day, then the SARS-CoV-2^Omicron EG.5.1^ was inoculated into the culture at MOI:100. Three hours post exposure, the virus was removed and the cells were washed out one time with culture medium and incubated with each drug solution. RNA-qPCR of the supernatant samples was performed after 3 days post viral exposure as described above.

To determine the cytotoxicity of each compound, cells were seeded in a 96-well plate (2 × 10^4^ cells/well). One day later, various concentrations of each compound were added, and cells were incubated for additional 3 days. The 50% cytotoxic concentrations (CC_50_) values were determined using the WST-8 assay and Cell Counting Kit-8 (Dojindo, Kumamoto, Japan).

### Immunocytochemistry

Cells in a 96-well microtiter culture plate were fixed with 4% paraformaldehyde–phosphate-buffered saline (PBS) for 15 min, washed with PBS (300 μL/well) three times for 5 min each time, and then blocked with a blocking buffer (10% goat serum, 1% bovine serum albumin [BSA], 0.3% Triton X-100, PBS 1x) for 1 h. After removal of the blocking buffer, the cells were immediately stained with a convalescent IgG fraction (concentration at 2.8 μg/mL), which was isolated from serum of a convalescent COVID-19 individual using a spin column-based antibody purification kit (Cosmo Bio, Tokyo, Japan) overnight at 4°C. The stained cells were washed with PBS (300 μL/well) three times for 5 min each time, and the cells were incubated with secondary antibody goat polyclonal antihuman IgG-Alexa Fluor 488 Fab fragment antibody (concentration at 2.5 μg/mL) (cat. no. 109-547-003) (Jackson ImmunoResearch Laboratories, Inc., West Grove, PA, USA), together with Texas Red-X dye-conjugated phalloidin (Thermo Fisher Scientific) for F-actin visualization for 2 h. After washing of the cells with PBS (300 μL/well) three times for 5 min each time, DAPI (4′,6-diamidino-2-phenylindole) solution (Thermo Fisher Scientific)-PBS (50 μL/well) was added to stain nuclei. Signals were acquired with a Cytation 5 cell imaging multimode reader (BioTek, Winooski, VT, USA).

### Determination of intracellular concentrations of compounds

The levels of intracellular concentrations of TKB245, TKB272, and nirmatrelvir, which represent the balance between the penetration through the membrane and intracellular degradation of the compounds, were determined in VeroE6 and HeLa^hACE2-TMPRSS2^ cells and the cell extracts were prepared as described previously (42). Three million cells were incubated with each drug (at 10, 50, and 100 μM final concentration) at 37°C for 6 h. Cells were harvested and washed with phosphate-buffered saline (PBS) three times, and cell pellets were resuspended in 70% methanol solution, and the suspensions were boiled at 95°C for 5 min with shaking. The boiled suspensions were cooled to room temperature and centrifuged at 16,440 × g for 10 min to separate cell debris from the solvent extract. Supernatants (solvent extracts) were transferred into new tubes, and the solvent was evaporated overnight. Dimethyl sulfoxide (DMSO) (50 μL per tube) was added to the dried tubes, and the tubes were incubated at 37°C for 1 h with shaking. The sample was solved with 50 µL solution (72% acetonitrile-0.05%TFA) and then analyzed using LC/MS/MS. 1 µL (TKB245 and TKB272) or 5 µL (nirmatrelvir) of prepared sample was injected and analyzed using a quadrupole-time-of-flight (QTOF) mass spectrometer equipped with a Captive Spray electrospray ionization platform in the positive mode (impact II, Bruker Daltonics) with liquid chromatography (Ultimate 3000 HPLC, Thermo Fisher scientific).Drugs were separated on an Acclaim PepMap 100 C18LC column (0.075 mm × 150 mm, 2 µm particle) using solvent A (water–0.1% formic acid [FA]) and solvent B (acetonitrile–0.1% FA). The flow rate was set to 300 nL/min, and the column was equilibrated with 95% solvent A and 5% solvent B. Following each injection, solvent B was increased to 95% over a 1 min period. 95% solvent B was kept for 8 min and then returned to starting conditions over the next 1 min. Each drug was detected by QTOF-MS using multiple reaction monitoring (MRM). In the MRM transition of TKB-272, -245, or -nirmatrelvir, the precursor ion and quantifier ion were, respectively, m/z654.2 and m/z110.1, m/z654.2 and m/z110.1, or m/z500.2 and m/z110.1. The amount of drug obtained in the extracts was determined by comparison to standards of each purified drug in 72% acetonitrile-0.05%TFA.The above-mentioned LC/MS analysis was performed complying with the community requirements by Alseekh et al. (43) and the detailed conditions are shown in source data.

### Animals

Jcl:ICR (ICR) female mice and PXB-mice (cDNA-uPA/SCID chimeric male mice with humanized liver with more than 70% of the liver replaced with human hepatocytes) were obtained from CLEA Japan (Tokyo, Japan) and PhoenixBio Co., Ltd. (Hiroshima, Japan), respectively. Hemizygous K18-hACE2 C57BL/6J mice (strain 2B6.Cg-Tg(K18-ACE2)2Prlmn/J) were obtained from the Jackson Laboratory, while C57BL/6JJcl female mice were purchased from CLEA Japan (Tokyo, Japan). All mice were housed in an air-conditioned animal room at 23 ± 5°C with a relative humidity of 55 ± 25°% under specific pathogen-free conditions, with a 12 h light/dark cycle (8 AM–8 PM/8 PM–8 AM). All mice were fed a standard rodent CRF-1 diet (Oriental yeast CO., LTD., Japan) and had ad libitum access to water. All animal experiments were approved by the Institutional Animal Care and Use Committees in NCGM (approval ID: no. 21057 and 2023-A050), the University of Tokyo (approval ID: PA19-72), and PhoenixBio Co., Ltd. (approval ID: 00038), and were carried out in accordance with institutional procedures, national guidelines and the relevant national laws on the protection of animals.

### Pharmacokinetic studies of TKB compounds in ICR mice, PXB-mice, and C57BL/6JJcl mice

For PK studies of TKB272, the ICR mice with body weights ranging from 20 to 25 g (*n* = 2) were intravenously (i.v.) or peroral (p.o.) administered 10 mg/kg of TKB272. At various time points after administration (0.25, 0.5, 1, 2, and 4 h), blood samples were collected from the retro-orbital venous plexus under sevoflurane anesthesia and centrifuged at 3,000 × g for 15 min to obtain plasma. For PK studies of TKB272 and nirmatelvir, the PXB mice with body weights ranging from 17 to 20 g (*n* = 3 per each group) were p.o. administered 10 mg/kg of TKB272 or nirmatelvir. At various time points after administration (0.167, 1, 4, 8, and 24 h), blood samples (∼30 μL/time point) were collected from the retro-orbital venous plexus under sevoflurane anesthesia using heparinized syringes and centrifuged at 3000 × g for 10 min to obtain plasma (∼12 μL/time point). All the antiviral agents studied in the present work were solubilized in saline containing 5% DMSO and 9.5% cremophor EL (Sigma-Aldrich, Co. LCC). PK studies in C57BL/6J mice, which are the background of K18 hACE mice, were also conducted treated with TKB272 (10 mg, 80 mg/kg, or 100 mg/kg each, solubilized in saline containing 20% DMSO and 9.5% cremophor EL) perorally (p.o.) and the time course of the plasma concentrations (4, 8, and 24 h) were examined (*n* = 3 per each experimental group) as described above.

### Antiviral efficacy against an XBB.1.5 variant

Nine-week-old female K18-hACE2 mice were used in this study. Baseline body weights were measured before infection. Under isoflurane anesthesia, mice were intranasally inoculated with XBB.1.5 at 1 × 10^4^ PFU/animal. Two hours post infection, these mice orally received TKB272 (50 mg/kg), TKB272 (50 mg/kg) plus ritonavir (3 mg/kg), nirmatrelvir (50 mg/kg) plus ritonavir (3 mg/kg), or vehicle twice daily. TKB272, nirmatrelvir and ritonavir were solubilized in saline containing 20% DMSO and 9.5% cremophor EL. For virological examinations, the animals were euthanized at 2 days post infection and the virus titers in the lungs were determined by plaque assays on VeroE6^TMPRSS2^ cells. All animal experiments with SARS-CoV-2 ^XBB.1.5^ were performed in animal biosafety level three (ABSL3) facility at the University of Tokyo.

### Statistical analyses of the changes in the viral loads in treated mice

GraphPad Prism (version 9.0.1) was utilized to examine all the data, and the Wilcoxon rank-sum test with two-sided was used for statistical analysis. The *P*-values and mean viral titers were presented and mean ± SD was plotted in the figure.

### Generation of a nirmatrelvir-resistant variant using SARS-CoV-2^WK-521^

VeroE6^TMPRSS2^ cells (10^5^/well seeded onto 24-well microtiter culture plates) were exposed to SARS-CoV-2^WK-521^ (MOI: 0.5) and cultured in the presence of nirmatrelvir or TKB272 at a half of initial EC_50_ dose. Viral replication was monitored by observing the cytopathic effect of the virus over the inverted microscope. The culture supernatants were harvested on days 3 or 4 and used at 1/100 dilution to infect fresh VeroE6^TMPRSS2^ cells for the next round of culture in the presence of increasing concentrations of nirmatrelvir or TKB272. When the virus propagated in the presence of the agent, the drug concentration was generally increased by 2-fold. RNA was extracted from virus populations including NIR-selected SARS-CoV-2^WK-521^ and was subjected to deep sequencing using Illumina COVIDseq with ARTIC V4.1 protocol and iSeq100 (Illumina, San Diego, CA). The obtained data were assembled using BaseSpace DRAGEN COVID Lineage v3.5.12. Mutation analysis was performed using Mutations Analysis Program (Stanford University, Coronavirus Antiviral & Resistance Database [CoVDB], https://covdb.stanford.edu/sierra/sars2/by-patterns/).

The entire genome of SARS-CoV-2 populations in question was sequenced and the NGS data have been deposited to DDBJ-Sequence Read Archive (DDBJ-DRA). The accession numbers for the NGS data of the selection-initiating virus and nirmatrelvir-selected virus are SRR27139898 and SRR27140505, respectively.

The full-genome nucleotide sequence of SARS-CoV-2 (Wuhan/Hu-1/2019) with or without the E166V mutation in the Nsp5 was assembled into the pBeloBAC11 vector to generate infectious cDNA clones under the control of a cytomegalovirus (CMV) promoter by using Gibson Assembly Master Mix (NEB) as described previously (44). To rescue the recombinant SARS-CoV-2, these BACs were transfected into HEK293T cells. At 3 days post-transfection, the supernatant was inoculated onto VeroE6/TMPRSS2 to prepare the virus stock. The virus titers of the stock viruses were determined by using plaque assays in VeroE6/TMPRSS2. The stock viruses were subjected to NGS as previously described (45) to confirm the absence of unwanted mutations.

All experiments were approved by the President of NCGM, the University of Tokyo, and Japanese Government, following consideration by the Institutional Committee of NCGM (approval ID: no. 2023-M020), the University of Tokyo (approval ID: 23-04), and Japanese Government (approval ID: 5-517), and were carried out in accordance with relevant guidelines.

### Protein expression and preparation

Both the sequence encoding wild-type SARS-CoV-2 M^pro^ (M^pro^_WT_) and single mutation introduced variant E166V(M^pro^_E166V)_ we cloned into the pGEX-4T1vector from Genscript Biotech (Piscataway, NJ). This construct featured a self-cleavage site at the N-terminus (SAVLQ/SGFRK) and at the C-terminus, a segment encoding the human rhinovirus 3 C PreScission protease cleavage site (SGVTFQ ↓ GP) followed by a His tag. These plasmid constructs were then transformed into BL21 Star (DE3) cells from Thermo Fisher Scientific (Waltham, MD). For optimal protein expression, cells were cultured in Terrific Broth media supplemented with ampicillin (Quality Biological, Gaithersburg, MD). Protein expression was induced when the optical density reached 0.8 at 600 nm by adding 1 mM isopropyl beta-D-thiogalactopyranoside (IPTG). The cultures were maintained at a temperature of 20°C overnight.

The SARS-CoV-2 M^pro^_WT_ and M^pro^_E166V_ protein was purified initially using affinity chromatography with TALON^TM^ cobalt-based affinity resin from Takara Bio (Shiga, Japan). The authentic N-terminus was generated through the 3CL M^pro^ autoprocessing mechanism during expression. Simultaneously, the authentic C-terminus was generated by treating the protein with PreScission protease. Subsequently, the resulting authentic 306 amino acid M^pro^ protein was further purified using size-exclusion chromatography (SEC) with a HiLoad Superdex 200 pg column from GE Healthcare. The SEC was performed in a buffer containing 20 mM Tris (pH 7.5), 150 mM NaCl, and 2 mM DTT. Finally, we obtained highly purified and concentrated SARS-CoV-2 M^pro^ protein.

### Native mass spectrometry

The M^pro^_WT_ and M^pro^_E166V_ substitution was diluted in 10 mM ammonium acetate (pH 6.7) to its final concentration of 7.5 µM and exposed to the respective M^pro^ inhibitor at a final concentration of 15 µM. Each sample solution was incubated at room temperature and introduced to the ESI-QTOF mass spectrometer (impact II, Bruker Daltonics Bremen, Germany) through an infusion pump at a flow rate of 3 μL/min. Samples were ionized in positive ion mode with following ion source parameters: dry heater: 200°C, nebulizer: 0.3 bar, dry gas: 3.0 L/min, capillary voltage: 4,500 V, end plate offset: −400 V, charging voltage: 2,000 V. MS scans have been acquired at a spectra rate of 1 Hz at a mass range from 500 to 6,000 m/z. Molecular masses by protein deconvolution were determined using DataAnalysis 4.4 (Bruker Daltonics, Bremen, Germany). All species detected by native MS including their theoretical molecular weights, experimental molecular weights, and mass errors are listed in Table S6. Relative abundance of each M^pro^ species was calculated by using sum of relative intensities of the each corresponding all charge state peaks.

### LC-MS/MS analysis

To the 5 µL of plasma sample, 20 µL of acetonitrile were added. The sample was stored for 15 min at 4°C to achieve optimal protein precipitation. After centrifugation at 15,000 × g at 4°C, the obtained supernatant was added trifluoroacetic acid to give a final concentration of 0.05% for LC-MS/MS analysis. To detect the compounds, analysis was done using a quadrupole-time-of-flight (QTOF) mass spectrometer equipped with a Captive Spray electro-spray ionization platform in the positive mode (impact II, Bruker Daltonics Bremen, Germany) with liquid chromatography (Ultimate 3000 HPLC, Thermo Fisher scientific). In total, 1 μL of prepared sample was injected and concentrated on an Acclaim PepMap100 C18 trap column (Thermo Fisher scientific) at flow rate of 20 μL/min. For sample separation, Acclaim PepMap 100 C18LC column (0.075 mm × 150 mm, 2 μm particle) (Thermo Fisher scientific) was used in conditions of isocratic mode of 95% acetonitrile 0.1% FA for 7 min, flow rate of 300 nL/min and temperature of 35°C. Compounds were approximately eluted at from 5 to 6 min. Following ion source parameters have been applied: Dry Heater: 150°C, Dry Gas: 8.0 L/min, Capillary voltage: 1,000 V, End plate offset: −500 V. Quantification was performed using multiple reaction monitoring (MRM). The MRM transitions from m/z654.2 to m/z110.1 and from m/z500.2 to m/z110.1 were used for TKB272 and nirmatrelvir, respectively. MS scans have been acquired at a spectra rate of 1 Hz at a mass range from 50 to 900 m/z. QuantAnalysis Ver2.2.1 (Bruker Daltonics, Bremen, Germany) was used for taking peak area of chromatogram of quantifier ions from MRM. The above-mentioned LC/MS analysis was performed complying with the community requirements by Alseekh et al. (43).

### Crystallization of M^pro^ in complex with TKB272

The M^pro^_WT_ and M^pro^_E166V_ was concentrated up to 6.6 mg/mL and incubated with 300 μM TKB272 or nirmatrelvir for 1 h before crystallization. Crystals were grown using hanging drop vapor diffusion method at 20°C. The reservoir solution contained 0.1 M HEPES pH 7.5, 5% polyethylene glycol (PEG) 6000 and 3% DMSO or 0.1 M MES pH 6.8, 15% PEG 6000 for M^pro^_WT_ and 0.1 M Tris–HCl pH 8.0, 22.5% PEG 6000, 3% DMSO or 0.1 M MES pH 6.2, 20% PEG 6000, 3% DMSO for M^pro^_E166V_. Crystals were soaked briefly in a cryoprotection solution (0.1 M HEPES pH 7.5, Tris–HCl pH 8.0 or 0.1 M MES pH 6.2 with 35% PEG 400 and 5% DMSO). X-ray data were collected at SPring-8 BL41XU (Hyōgo, Japan) and processed using DIALS using xia2 incorporated in CCP4i2 (46). The source wavelength for the data collection was 1.0 Å. Data collection statistics are shown in Table S7. The phase problem was solved by molecular replacement using MolRep (47) using the 1.25 Å structure of M^pro^ (PDB ID: 8DOX) as a model. All water molecules and ligand atoms were omitted from the starting model. Subsequent cycles of refinement were performed in REFMAC5 (48). Structure file of TKB272 were generated using the Dundee PRODRG2 server (49) and manually fitted to the electron density. All structural figures were produced with PyMOL (50) and UCSF Chimera (51).

### In vitro evaluation of genotoxic potential of TKB272

In order to examine the genetic mutagenesis of TKB272, a bacterial reverse mutation assay (“micro-Ames” bacterial mutation test) was conducted using four *Salmonella typhimurium* strains (TA100, TA1535, TA98, TA1537) and one *Escherichia coli* strain (WP2 uvrA). The preincubation method of the μAmes test using a 24-well plate was performed under metabolic activation with S9 mixture (S9 consists of rat liver homogenate S9 fraction and coenzyme) and nonmetabolic activation conditions (52). The study was conducted at a total of 8 doses of 111, 37.0, 12.3, 4.12, 1.37, 0.457, and 0.152 µg/well with the highest dose of 333 µg/well. Dimethyl sulfoxide (DMSO) was used as the solvent for the test substance and placed as a negative control of the present evaluation (BOZO research center Inc., Tokyo, Japan).

## Supplementary Material

pgae578_Supplementary_Data

## Data Availability

Crystal structure data that support the findings of this study have been deposited in Protein Data Bank with the PDB IDs: 8UH5, 8UH9, and 8UH8. All study data are included in the article and/or SI Appendix.
