# Supplementary material for: An orally available P1′-5-fluorinated Mpro inhibitor blocks SARS-CoV-2 replication without booster and exhibits high genetic barrier
Source: PNAS Nexus. 2025 Jan 7;4(1):pgae578. doi: 10.1093/pnasnexus/pgae578 (PMC11740726; doi:10.1093/pnasnexus/pgae578)
Supplement: pgae578_Supplementary_Data [file pgae578_supplementary_data.pdf]

PNAS nexus

Supplementary information for

An orally available P1'-5-fluorinated M^{pro} inhibitor blocks SARS-CoV-2 replication without booster and exhibits high genetic barrier

Nobuyo Higashi-Kuwata[1*], Haydar Bulut[2], Hironori Hayashi[3], Kohei Tsuji[4], Hiromi Ogata-Aoki[1,5,6], Maki Kiso[7], Nobutoki Takamune[8], Naoki Kishimoto[8], Shin-ichiro Hattori[1], Takahiro Ishii[4], Takuya Kobayakawa[4], Kenta Nakano[9], Yukiko Shimizu[9], Debananda Das[2], Junji Saruwatari[10], Kazuya Hasegawa[11], Kazutaka Murayama[12], Yoshikazu Sukenaga[1], Yuki Takamatsu[1], Kazuhisa Yoshimura[13], Manabu Aoki[1,14], Yuri Furusawa[15,16], Tadashi Okamura[9], Seiya Yamayoshi[7,16], Yoshihiro Kawaoka[7,16,17], Shogo Misumi[8], Hirokazu Tamamura[4], and Hiroaki Mitsuya[1,2,5*]

[1]Department of Refractory Viral Diseases, National Center for Global Health and Medicine Research Institute, Shinjuku-ku, Tokyo 162-8655, Japan; [2]Experimental Retrovirology Section, HIV and AIDS Malignancy Branch, National Cancer Institute, NIH, Bethesda, MD20892, USA; [3]Department of Infectious Diseases, International Research Institute of Disaster Science, Tohoku University, Aoba-ku, Sendai 980-8572, Japan; [4]Department of Medicinal Chemistry, Institute of Biomaterials and Bioengineering, Tokyo Medical and Dental University, Chiyoda-ku, Tokyo 101-0062, Japan; [5]Kumamoto University Hospital, Kumamoto, 860-8556, Japan; [6]Division of Hematopoiesis, Joint Research Center for Human Retrovirus Infection & Graduate School of Medical Sciences, Kumamoto University, Kumamoto, 860-0811; [7]Division of Virology, Institute of Medical Science, University of Tokyo, Tokyo108-8639, Japan; [8]Department of Environmental and Molecular Health Sciences, Faculty of Life Sciences, Kumamoto University, Chuo-ku, Kumamoto 862-0973, Japan; [9]Department of Laboratory Animal Medicine, Research Institute, National Center for Global Health and Medicine (NCGM), Tokyo 162-8655, Japan; [10]Division of Pharmacology and Therapeutics, Graduate School of Pharmaceutical Sciences, Kumamoto University, Chuo-ku, Kumamoto 862-0973, Japan; [11]Structural Biology Division, Japan Synchrotron Radiation Research Institute, Hyogo, Japan; [12]Graduate School of Biomedical Engineering, Tohoku University, Miyagi, Japan; [13]Tokyo Metropolitan Institute of Public Health, Tokyo, Japan; [14]Department of Medical Technology, Kumamoto Health Science University, Kumamoto, Japan; [15]Division of Virology, Institute of Medical Science, University of Tokyo, Tokyo, Japan; [16]The Research Center for Global Viral Diseases, National Center for Global Health and Medicine Research Institute, Tokyo, Japan; [17]Influenza Research Institute, Department of Pathobiological Sciences, School of Veterinary Medicine, University of Wisconsin-Madison, Madison, WI53711, USA

* **Corresponding authors:** Hiroaki Mitsuya and Nobuyo Higashi-Kuwata

Emails: hmitsuya@hosp.ncgm.go.jp; hiroaki.mitsuya2@nih.gov; and nkuwata@ri.ncgm.go.jp

This PDF file includes:

Figures S1 to S8
Tables S1 to S7

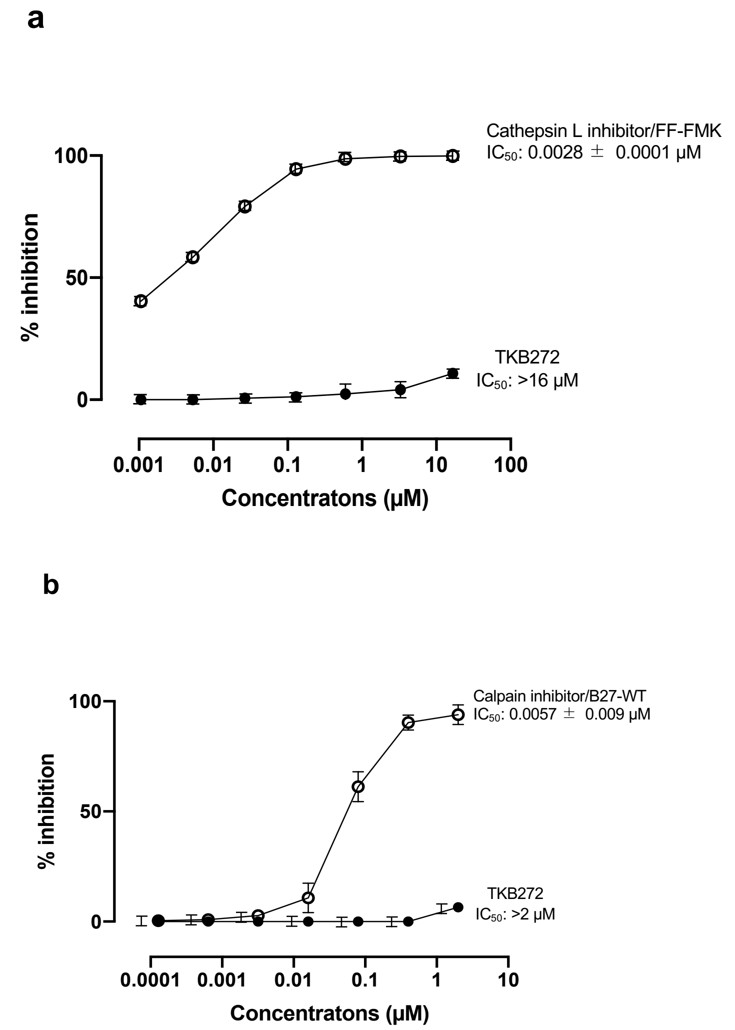

Fig. S1. Inhibition of human cysteine proteases, cathepsin L and calpains, by TKB272. a. The inhibition curve of human cathepsin L by TKB272, or FF-FMK after a 30-minute incubation of the enzyme with increasing inhibitor concentrations. **b.** The inhibition curve of human calpains by TKB272 or B27-WT after a 60-minute incubation of the enzyme with increasing inhibitor concentrations. FF-FMK and B27-WT served as positive controls for human cathepsin L inhibitor and human calpain inhibitor, respectively. The data are shown with error bars in panels a and b. The mean IC_{50} values ± 1 S.D. of the assay results determined in triplicate (n=3).

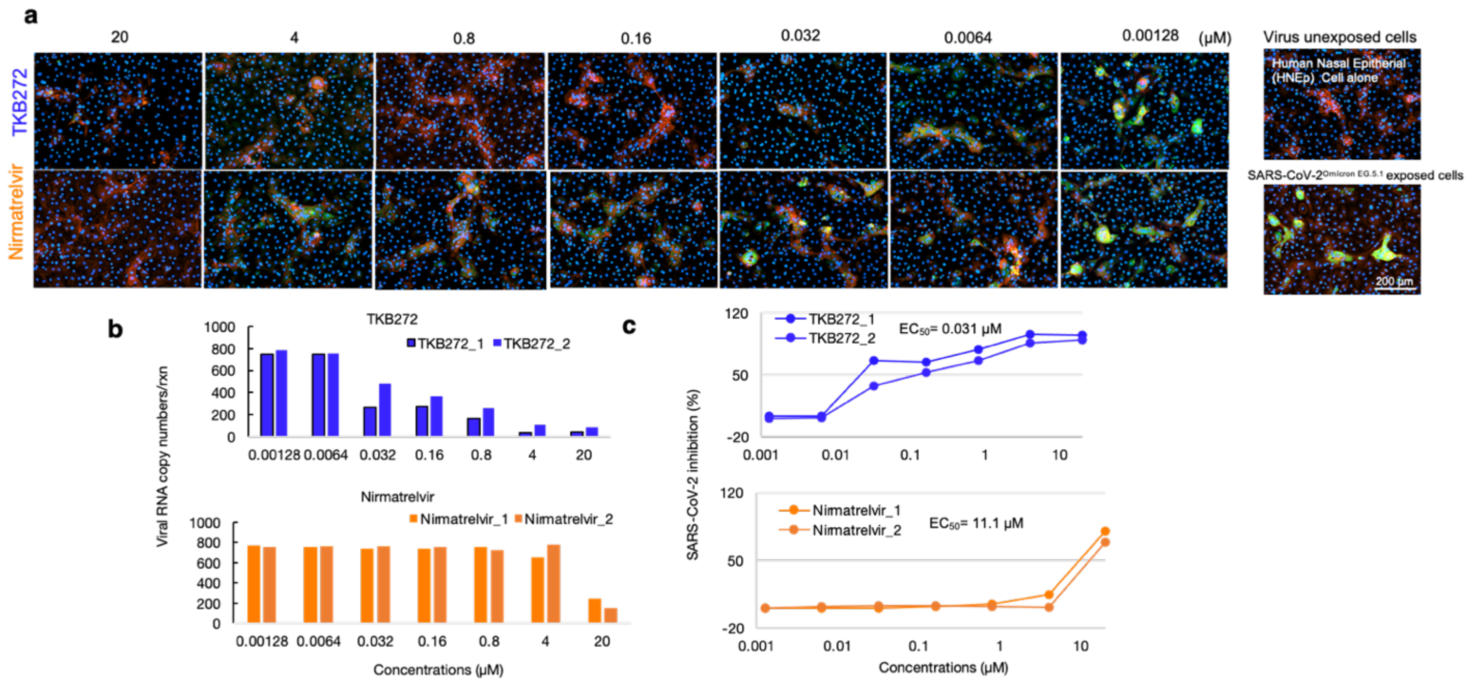

Fig. S2. TKB272 demonstrates potent anti-SARS-CoV-2$^{\text{Omicron EG.5.1}}$ activity in primary human nasal epithelial (HNEp) cells. a Immunocytochemistry images of cells cultured alone (the very right top panel), SARS-CoV-2$^{\text{Omicron EG.5.1}}$-exposed (MOI:100) cells cultured in the absence of compound (the very right bottom panel), virus-exposed and cultured in the presence of TKB272 (top row panels) or nirmatrelvir (bottom row panels) are shown. SARS-CoV-2 antigens, F-actin, and nuclei are in dicated in green, red, and blue, respectively. Comparative results were obtained in 2 independently conducted experiments and representative images are shown. **b** Viral RNA copy numbers in the culture supernatants in the presence of TKB272 (upper) and nirmatrelvir (lower). **c** Percentage inhibition of SARS-CoV-2 in the presence of TKB272 (upper) and nirmatrelvir (lower) and EC_{50} values are shown.

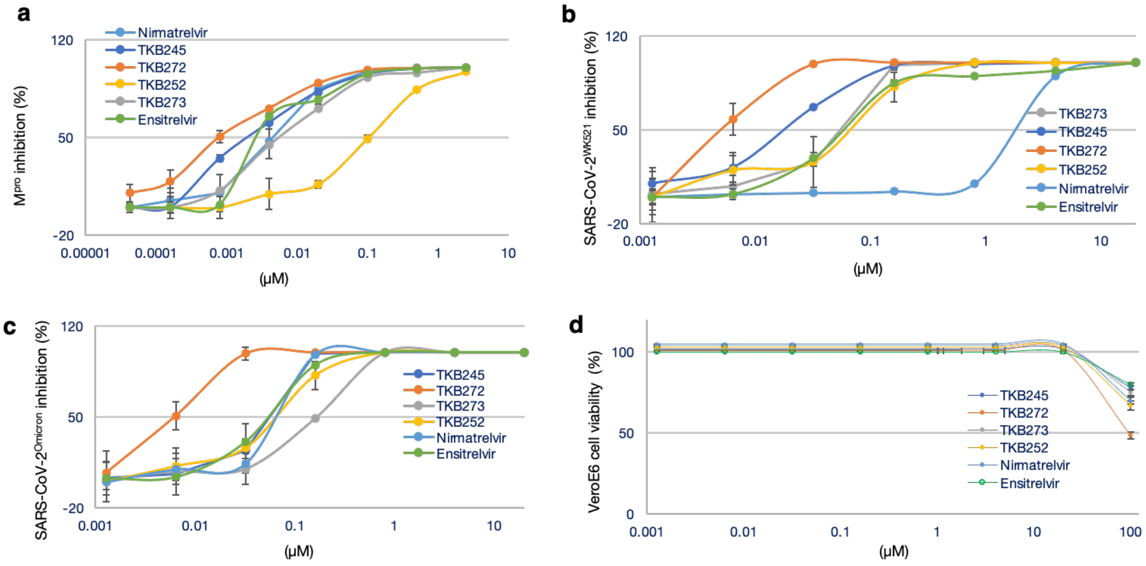

Fig. S3. M^{pro} enzymatic assay, cell-based anti-SARS-CoV-2 assay, and cytotoxicity inhibition assay data. Each curve demonstrates **a** fifty percent inhibition concentration (IC_{50}) in cell-free M^{pro} enzyme assay (**b** and **c)**, fifty percent effective concentrations (EC_{50}) in cell-based assay, and **d** fifty percent cytotoxic concentrations (CC_{50}) summarized in Table 1. Representative curves from three independent assays are shown. Error bars denote arithmetic means ± 1 S.D.

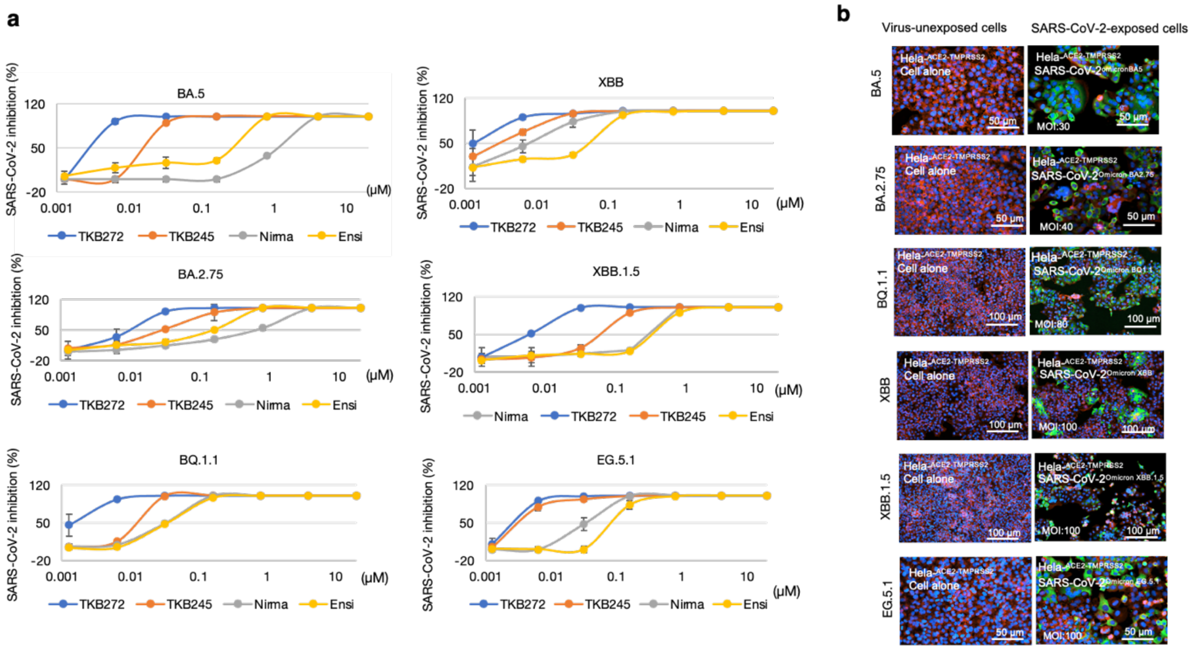

Fig. S4. Cell-based anti-SARS-CoV-2 (6 different Omicron variants) assay curves and immunocytochemistry images of cells infected with the Omicron variants summarized in Table 2. (**a**) Percent inhibition curves of 6 different SARS-CoV-2 omicron variants obtained in the presence of compounds are shown. Representative curves from three independent assays are shown. Error bars denote arithmetic means ± 1 S.D. Note that, in cell-based antiviral assays, daughter virions produced from infected target cells spread within the adherent cell culture and such spread is affected by the nature of spike proteins and other inherent virally-coded functions and structures of each specific variants. Thus, in the cell-based assays especially using adherent target cells, certain levels of variability in the assay results (such as EC_{50} values) are seen. (**b**) Immunocytochemistry images of cells cultured alone (left row), SARS-CoV-2 Omicron variant-exposed cells cultured in the absence of compound (right row), each MOI value is shown in each panel. SARS-CoV-2 antigens, F-actin, and nuclei are indicated in green, red, and blue, respectively. Representative images from 3 independent experiments are shown.

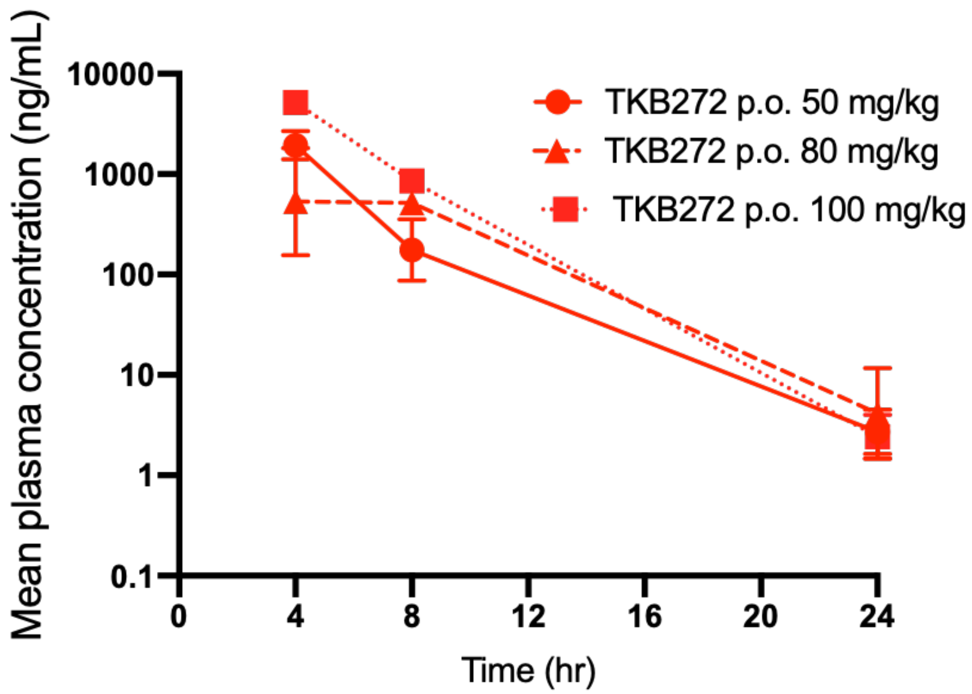

Fig. S5. Pharmacokinetic profiles of TKB272 in C57BL/6J mice. The C57BL/6J mice are the background of K18 hACE mice were treated with TKB272 (10 mg, 80mg/kg, or 100mg/kg each) perorally (p.o.) and the time course of the plasma concentrations were examined (n = 3 per each experimental group). Representative curves from three independent assays are shown. Error bars denote geometric means ± 1 S.D.

a b

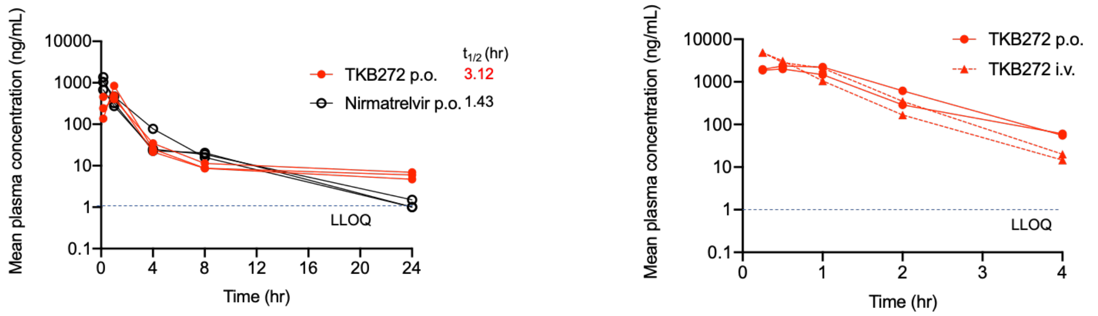

c

PK parameters	i.v.	p.o..
t1/2(h)	0.5 ± 0.03	3.4 ± 0.3
AUC (μg/mL・hr)	4.0 ± 0.4	4.2 ± 0.4
CL (mL/hr/kg)	63.3 ± 6.7	63.7 ± 5.8
Vdss (mL/kg)	48.5 ± 3.6	310.0± 52.6
F [%]	99.3	

Fig. S6. Pharmacokinetic profiles of TKB272. a Human liver-chimeric mice (PXB-mice) were treated with TKB272 or nirmatrelvir (10 mg/kg each) perorally (p.o.) and the time course of the plasma concentrations were examined (n = 3 per each experimental group). b Pharmacokinetics in ICR mice were determined with TKB272 (10 mg/kg) intravascularly (i.v.) or peroral (p.o.), and time course of the plasma concentrations was plotted. (n = 2 per each experimental group). c Summary of pharmacokinetic parameters of TKB272 in ICR mice (n = 2). AUC, area under the concentration-time curve; CL, clearance; F, oral bioavailability; $t_{1/2}$, half-life; Vdss, distribution volume. Data are presented mean ± S.E. The lower limit of quantification (LLOQ) for each compound was determined to be 1.0 ng/mL.

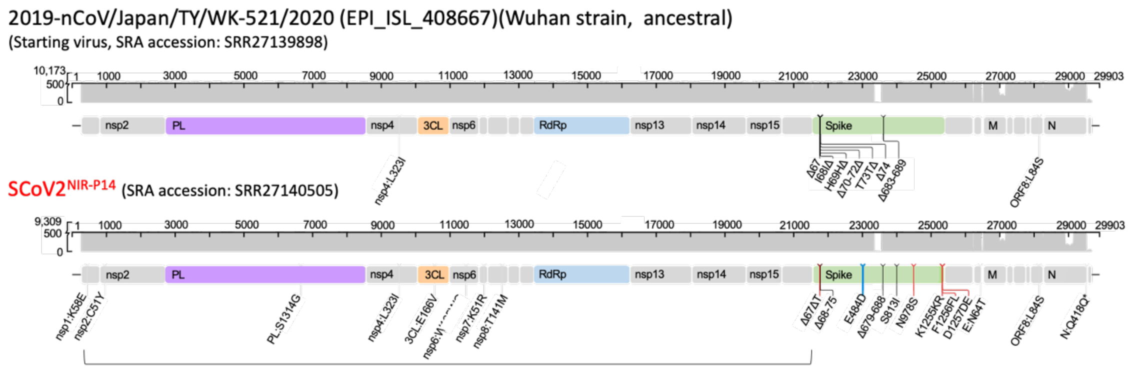

Gene-position	WK-521	NIR-P14	%
Nsp1-58	K	E	99.75
Nsp2-51	C	Y	95.54
PLpro-1314	S	G	99.89
Mpro-166	E	V	99.76
Nsp6-165	W	G	29.68
Nsp7-51	K	R	99.91
Nsp8-141	T	M	99.83

Fig. S7. NGS analysis RNA extracted from the primary SCoV2^{WK-521} and NIR-14-passaged SCoV2^{WK-521} (SCoV2$^{NIR-P14}$) was subjected to next-generation sequencing using Illumina COVIDseq with the ARTIC V4.1 protocol and iSeq100. The data obtained were assembled using BaseSpace DRAGEN COVID Lineage v3.5.12. Mutation analysis was performed using Mutations Analysis Program (Stanford University, Coronavirus Antiviral & Resistance Database, https://covdb.stanford.edu/sierra/sars2/by-patterns/).

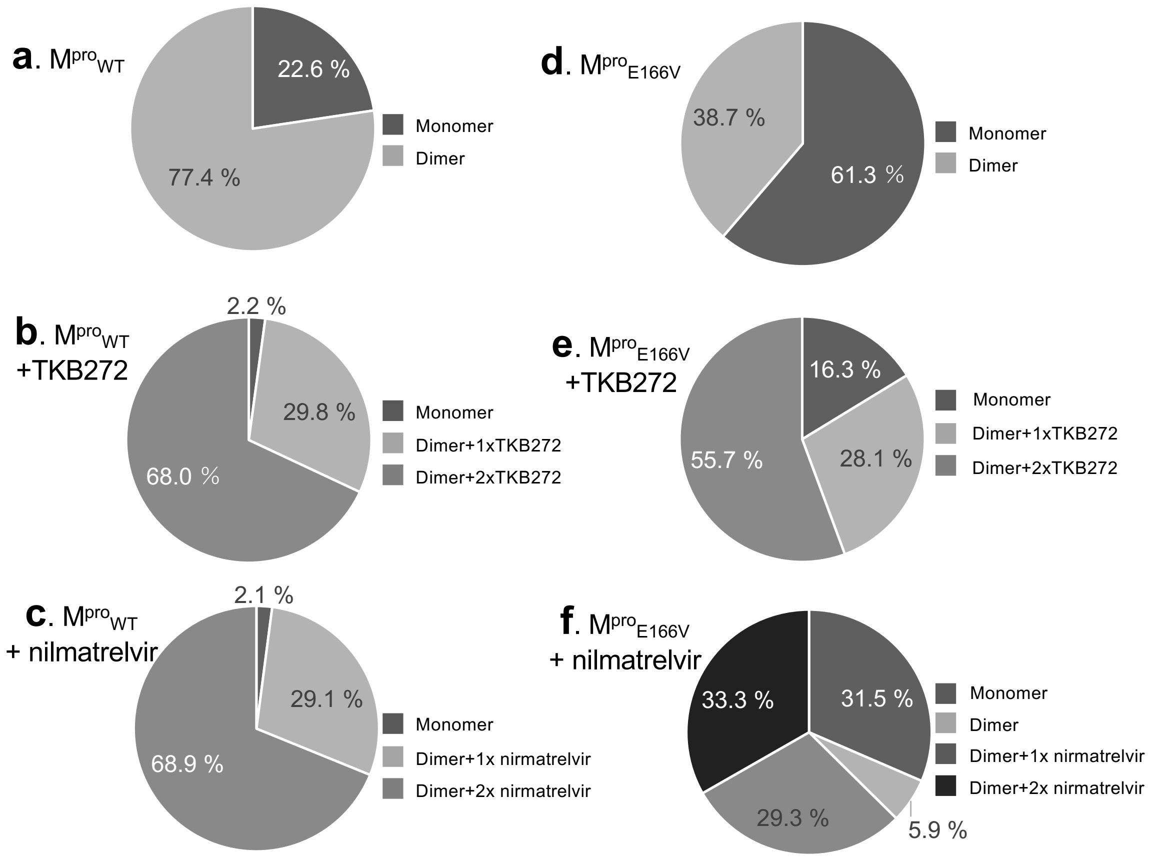

Fig. S8. Peak ratios of M^{pro}, M^{pro}-inhibitor complex, M^{pro}_{WT}- and M^{pro}_{E166V}-inhibitor complex observed by native MS. The peak ratios were calculated for each spectrum by integrating the relevant peak intensity from all charge states.

Table S1. TKB272 demonstrates more potent SARS-CoV-2^{WK-521} antiviral activity in the presence of P-glycoprotein inhibitor, CP-100356, than in the absence of the inhibitor.

Cell line	EC_{50} (μM)		
	TKB245	TKB272	Nirmatrelvir
VeroE6	0.033 ± 0.016	0.009 ± 0.006	1.33 ± 0.23
VeroE6 + 2μM CP-100356	0.0035 ± 0.0011	0.0004 ± 0.0001	0.0363 ± 0.0045

The P-glycoprotein inhibitor, CP-100356 (efflux inhibitor, EI, 2 μM), was added to inhibit the P-glycoprotein–mediated efflux of compounds in VeroE6 cells. Anti-SARS-CoV-2 activity of compounds discussed in the present study was determined with RT-qPCR of viral RNA from culture medium of SARS-CoV2-exposed cells. Each EC_{50} value was calculated as previously published[11]. Data from three independent assays are shown as arithmetic means ± 1 S.D.

Table S2. Effect of the presence of hAAG on the antiviral activity of TKB272.

hAAG (mg/mL)	EC_{50} (μM)	
	TKB272	Nirmatrelvir
0	0.003 ± 0.002	0.021 ± 0.003
1	0.027 ± 0.009	0.157 ± 0.045
5	0.293 ± 0.074	1.583 ± 0.629

Human aipha-1-acid glycoprotein (*hAAG), which is one of human plasma proteins and functions as a carrier for basic or neutral lipophilic compounds and is known as a major carrier of basic (positively charged) drugs, steroids, and protease inhibitors. The physiological concentrations of hAAG range 0.6-1.2 mg/mL Anti-SARS-CoV-2 activity of TKB272 and nirmatrelvir was evaluated with RT-qPCR of viral RNA from culture medium of SARS-CoV2$^{XBB1.5}$-exposed HeLa$^{hACE2-TMPRSS2}$ cells. Each EC_{50} value was calculated as previously published[11]. Data from three independent assays are shown as arithmetic means ± 1 S.D.

Table S3. Inhibition of SARS-CoV-2 carrying E166V substitution by ensitrelvir and molnupiravir in VeroE6 cells in the presence of CP-100356.

a

Compounds	EC_{50} (μM)	
	SARS-CoV-2^{WK-521}	SARS-CoV-2$^{E166V-P14}$
Ensitrelvir	0.03 ± 0.01	0.54 ± 0.17 (x18)
Molnupiravir	0.75 ± 0.20	0.84 ± 0.10 (x1.12)

b

Compounds	EC_{50} (μM)	
	rgSARS-CoV-2^{WT}	rgSARS-CoV-2^{E166V}
Ensitrelvir	0.024 ± 0.007	0.311 ± 0.147 (x12)
Molnupiravir	0.418 ± 0.290	0.368 ± 0.051 (x0.8)

Panel a. A P-glycoprotein inhibitor, CP-100356 (efflux inhibitor, 2 μM), was added to inhibit the P-glycoprotein–mediated efflux of compounds in VeroE6 cells. Anti-SARS-CoV-2 activity of the compounds was evaluated with RT-qPCR of viral RNA extracted from culture medium of the SARS-CoV2^{WK-521}- or SARS-CoV2$^{E166V-P14}$-exposed VeroE6 cells. EC_{50} value was calculated as previously published[11]. Panel b. Antiviral activity of ensitrelvir and molnupiravir against recombinant infectious SARS-CoV-2 with or without E166V substitution (rgSARS-CoV-2^{WT} and rgSARS-CoV-2^{E166V}) in the presence of CP-100356 is shown. Data from three independent assays are shown as means ± 1 S.D. Values shown in parentheses denote fold changes of the mean EC_{50} values relative to their inhibition of rgSARS-CoV-2^{WT} obtained from three independent experiments.

Table S4. Results of the bacterial reverse mutation study of TKB272

With (+) or without (–) S9 mix	Dose level (µg / well)	Number of revertants (number of colonies / plate)				
		Base-pair change type			Frameshift type	
		TA100	TA1535	WP2*uvrA*	TA98	TA1537
S9 mix (–)	Negative control (DMSO)	8.3 ± 2.9	0.4 ± 0.7	1.3 ± 1.0	1.5 ± 1.2	0. 3± 0.5
	0.152	6.0 ± 1.0	0.7 ± 1.2	1.0 ± 1.0	0.3 ± 0.6	0.3 ± 0.6
	0.457	4.3 ± 0.6	0.3 ± 0.6	2.7 ± 1.5	2.7 ± 1.5	0.0 ± 0.0
	1.37	7.3 ± 0.6	0.3 ± 0.6	2.3 ± 1.5	2.3 ± 0.6	0.7 ± 0.6
	4.12	6.0 ± 1.0	0.7 ± 0.6	2.0 ± 2.0	1.7 ± 0.6	0.3 ± 0.6
	12.3	8.3 ± 4.2	0.7 ± 0.6	1.3 ± 1.2	1.3 ± 0.6	0.7 ± 1.2
	37.0*	5.0 ± 1.7	0.0 ± 0.0	2.3 ± 1.2	2.3 ± 1.5	0.0 ± 0.0
	111*	6.0 ± 2.0	0.3 ± 0.6	1.3 ± 1.5	1.3 ± 0.6	0.0 ± 0.0
	333*	7.3 ± 2.1	0.3 ± 0.6	2.0 ± 0.0	0.7 ± 0.6	0.3 ± 0.6
S9 mix (+)	Negative control (DMSO)	7.7 ± 2.0	0.7 ± 0.7	2.0 ± 1.5	1.8 ± 1.1	0.3 ± 0.6
	0.152	8.3 ± 2.1	0.7 ± 1.2	1.0± 1.0	2.7 ± 0.6	0.3 ± 0.6
	0.457	6.7 ± 2.1	0.3 ± 0.6	1.3 ± 0.6	1.7 ± 1.2	0.3 ± 0.6
	1.37	7.0 ± 1.	0.7 ± 0.6	1.0± 1.0	1.3 ± 0.6	0.7 ± 0.6
	4.12	8.3 ± 0.6	0.7 ± 0.6	1.3 ± 0.6	2.0 ± 1.0	0.3 ± 0.6
	12.3	7.0 ± 3.6	0.7 ± 0.6	1.3 ± 1.2	2.0 ± 0.0	0.3 ± 0.6
	37.0	8.7 ± 2.5	0.7 ± 1.2	1.7 ± 1.2	2.0 ± 1.0	0.0 ± 0.0
	111*	7.0 ± 1.7	1.0 ± 1.0	1.7 ± 0.6	2.0 ± 2.0	0.7 ± 0.6
	333*	6.3 ± 1.2	0.3 ± 0.6	2.0 ± 1.0	2.0 ± 1.7	0.3 ± 0.6
Positive control S9 mix (–)	Name	AF-2	NaN3	AF -2	AF-2	ICR-191
	Dose (ng/well)	0.667	33.3	0.667	6.67	66.7
	Number of colonies / plate	29.0 ± 4.	29.3 ± 9.1	10.7± 3.1	22.7 ± 3.1	37.3± 2.5
Positive control S9 mix (+)	Name	B[a]P	2AA	2AA	B[a]P	B[a]P
	Dose (ng/well)	333	133	667	333	333
	Number of colonies / plate	40.7 ± 4.2	15.3 ± 1.2	49.0± 7.9	20.3± 2.1	6.0± 2.6

Note: *: Precipitation was observed. Values represent mean and standard deviation of triplicate plates (mean ±S.D.) Negative control: Dimethyl sulfoxide Positive controls: AF-2:2-(2-Furyl)-3-(5-nitro-2-furyl)acrylamide, NaN3 (SodiumAzide), ICR-191:2-Methoxy-6-chloro-9-[3-(2-chloroethyl)-aminopropylamino] acridine·2HCl, B[a]P: Benzo[a]pyrene, 2-AA: 2-Aminoanthracene

Table S5. A list of SARS-CoV-2 variants used in this study

Virus Name	GISAID Accession ID	Pango lineage/Variant
hCoV-19/Japan/ TY-WK-521/2020	EPI_ISL_408667	A
hCoV-19/Japan/ TY41-716/2022	EPI_ISL_13969765	BA.2.75
hCoV-19/Japan/23-018-P1/2022	EPI_ISL_16889601	XBB.1.5
hCoV-19/USA/MD-HP40900-PIDYSWHNUB/2022	EPI_ISL_16026423	XBB.1.5
hCoV-19/Japan/TKYTS14631/2022	EPI_ISL_12812500.1	BA.5
hCoV-19/Japan/TY41-795/2022	EPI_ISL_15669344	XBB
hCoV-19/Japan/TY41-796/2022	EPI_ISL_15579783	BQ.1.1
hCoV-19/Japan/TKYnat14564/2023	EPI_ISL_18082364	EG.5.1

Table S6. Species of M^{pro} or M^{pro}-inhibitor complexes observed by native MS.

Species of M^{pro}_{WT}	Theoretical mass	Experimental mass*	Mass error
M^{pro} (monomer)	33796.6	33795.5	-1.1
2xM^{pro} (dimer)	67593.3	67596.0	2.7
2xM^{pro} + 1xTKB272	68247.0	68255.0	8.0
2xM^{pro} + 2xTKB272	68900.6	68907.7	7.1
2xM^{pro} + 1xNirmatrelvir	68092.8	68089.1	-3.7
2xM^{pro} + 2xNirmatrelvir	68592.3	68592.2	-0.1

Species of M^{pro}_{E166V}	Theoretical mass	Experimental mass*	Mass error
M^{pro} (monomer)	33766.7	33765.9	-0.8
2xM^{pro} (dimer)	67533.3	67531.2	-2.1
2xM^{pro} + 1xTKB272	68187.0	68191.3	4.3
2xM^{pro} + 2xTKB272	68840.7	68831.1	-9.6
2xM^{pro} + 1xNirmatrelvir	68032.8	68028.0	-4.8
2xM^{pro} + 2xNirmatrelvir	68532.4	68530.2	-2.2

Species of M^{pro} or M^{pro}-inhibitor complexes observed in native MS, as shown Figs. 6a and 6b, were identified by comparing each deconvoluted mass from the measured spectra to the corresponding theoretical mass. *Mean values of deconvoluted masses determined using at least three charge states.

Table S7. Data collection and refinement statistics (molecular replacement)

	M^{pro}_{WT} TKB272(8UH5)	M^{pro}_{E166V} TKB272 (8UH9)	M^{pro}_{E166V} Apo (8UH8)
**Data collection**			
Space group	C 1 2 1	C 1 2 1	C 1 2 1
Cell dimensions			
a, *b*, *c* (Ǎ)	114.00, 53.07, 45.78	113.67, 53.09, 45.77	13.07, 53.76, 44.64
α, β, γ (°)	90.00, 102.17, 90.00	90.00, 101.96, 90.00	90 100.911 90
Resolution (Ǎ)	55.72 - 1.903 (1.971 - 1.903) *	47.91 - 2.07 (2.144 - 2.07)*	55.51 - 1.801 (1.865 - 1.801)
R_{sym} or R_{merge}	0.07032 (1.049)	0.1795 (2.927)	0.04858 (0.5078)
$I / \sigma I$	15.49 (1.02)	9.16 (1.05)	20.14 (1.30)
Completeness (%)	99.85 (99.19)	97.39 (76.57)	97.91 (96.28)
Redundancy	8.5 (8.6)	6.9 (6.7)	7.1 (7.3)
**Refinement**			
Resolution (Ǎ)	55.72 - 1.903	56.52 - 2.07	55.51 - 1.801
No. reflections	21114 / 1084	16461 / 833	23993 / 1182
R_{work} / R_{free}	0.189 / 0.234	0.196 / 0.271	0.212 / 0.265
No. atoms	2444	2427	2421
Protein	2367	2365	2365
Ligand/ion	52	56	0
Water	25	6	56
B-factors	46.51	60.35	41.93
Protein	46.62	60.17	42.06
Ligand/ion	44.72	69.94	
Water	39.84	43.70	36.32
R.m.s. deviations			
Bond lengths (Ǎ)	0.015	0.0075	0.011
Bond angles (°)	1.92	1.363	1.75

*Values in parentheses are for the highest-resolution shell.
